# The endogenous *mex-3* 3´UTR is required for germline repression and contributes to optimal fecundity in *C*. *elegans*

**DOI:** 10.1371/journal.pgen.1009775

**Published:** 2021-08-23

**Authors:** Mennatallah M. Y. Albarqi, Sean P. Ryder

**Affiliations:** Department of Biochemistry and Molecular Pharmacology, University of Massachusetts Medical School, Worcester, Massachusetts, United States of America; The University of North Carolina at Chapel Hill, UNITED STATES

## Abstract

RNA regulation is essential to successful reproduction. Messenger RNAs delivered from parent to progeny govern early embryonic development. RNA-binding proteins (RBPs) are the key effectors of this process, regulating the translation and stability of parental transcripts to control cell fate specification events prior to zygotic gene activation. The KH-domain RBP MEX-3 is conserved from nematode to human. It was first discovered in *Caenorhabditis elegans*, where it is essential for anterior cell fate and embryo viability. Here, we show that loss of the endogenous *mex-3* 3´UTR disrupts its germline expression pattern. An allelic series of 3´UTR deletion variants identify repressing regions of the UTR and demonstrate that repression is not precisely coupled to reproductive success. We also show that several RBPs regulate *mex-3* mRNA through its 3´UTR to define its unique germline spatiotemporal expression pattern. Additionally, we find that both poly(A) tail length control and the translation initiation factor IFE-3 contribute to its expression pattern. Together, our results establish the importance of the *mex-3* 3´UTR to reproductive health and its expression in the germline. Our results suggest that additional mechanisms control MEX-3 function when 3´UTR regulation is compromised.

## Author summary

In sexually reproducing organisms, germ cells undergo meiosis and differentiate to form oocytes or sperm. Coordination of this process requires a gene regulatory program that acts while the genome is undergoing chromatin condensation. As such, RNA regulatory pathways are an important contributor. The germline of the nematode *Caenorhabditis elegans* is a suitable model system to study germ cell differentiation. Several RNA-binding proteins (RBPs) coordinate each transition in the germline such as the transition from mitosis to meiosis. MEX-3 is a conserved RNA-binding protein found in most animals including humans. In *C*. *elegans*, MEX-3 displays a highly restricted pattern of expression. Here, we define the importance of the 3´UTR in regulating MEX-3 expression pattern *in vivo* and characterize the RNA-binding proteins involved in this regulation. Our results show that deleting various *mex-3* 3´UTR regions alter the pattern of expression in the germline in various ways. These mutations also reduced—but did not eliminate—reproductive capacity. Finally, we demonstrate that multiple post-transcriptional mechanisms control MEX-3 levels in different domains of the germline. Our data suggest that coordination of MEX-3 activity requires multiple layers of regulation to ensure reproductive robustness.

## Introduction

Regulation of mRNA metabolism occurs in all cells in all kingdoms of life. In the nucleus, pre-mRNA undergoes splicing, 5´-capping, and 3´-end cleavage and polyadenylation [1]. Once mature, mRNA is exported to the cytoplasm where it undergoes further post-transcriptional modifications prior to translation. In the cytoplasm, mRNA can be stabilized by additional poly-adenylation or targeted for degradation by exonucleases through deadenylation and de-capping [2,3]. This layer of regulation contributes to the amount of protein produced per transcript. Failure to properly process the pre-mRNA in the nucleus or the mature mRNA in the cytoplasm can lead to dysregulation of protein production and disease. Post-transcriptional regulation is especially critical in developmental processes such as gametogenesis and embryogenesis [4,5]. During the early stages of embryogenesis prior to the onset of zygotic transcription, inherited maternal mRNAs and proteins are essential to axis determination and cell fate specification [6–8]. Maternal mRNAs must be produced in the germline, packaged into oocytes, silenced, activated at the right time and place in the embryo, and then cleared once zygotic transcription begins. Accordingly, a variety of post-transcriptional regulatory mechanisms are required to coordinate this developmental program. Much remains to be learned about how they collaborate to achieve distinct spatiotemporal expression patterns for different maternal mRNAs.

**Fig 1:**
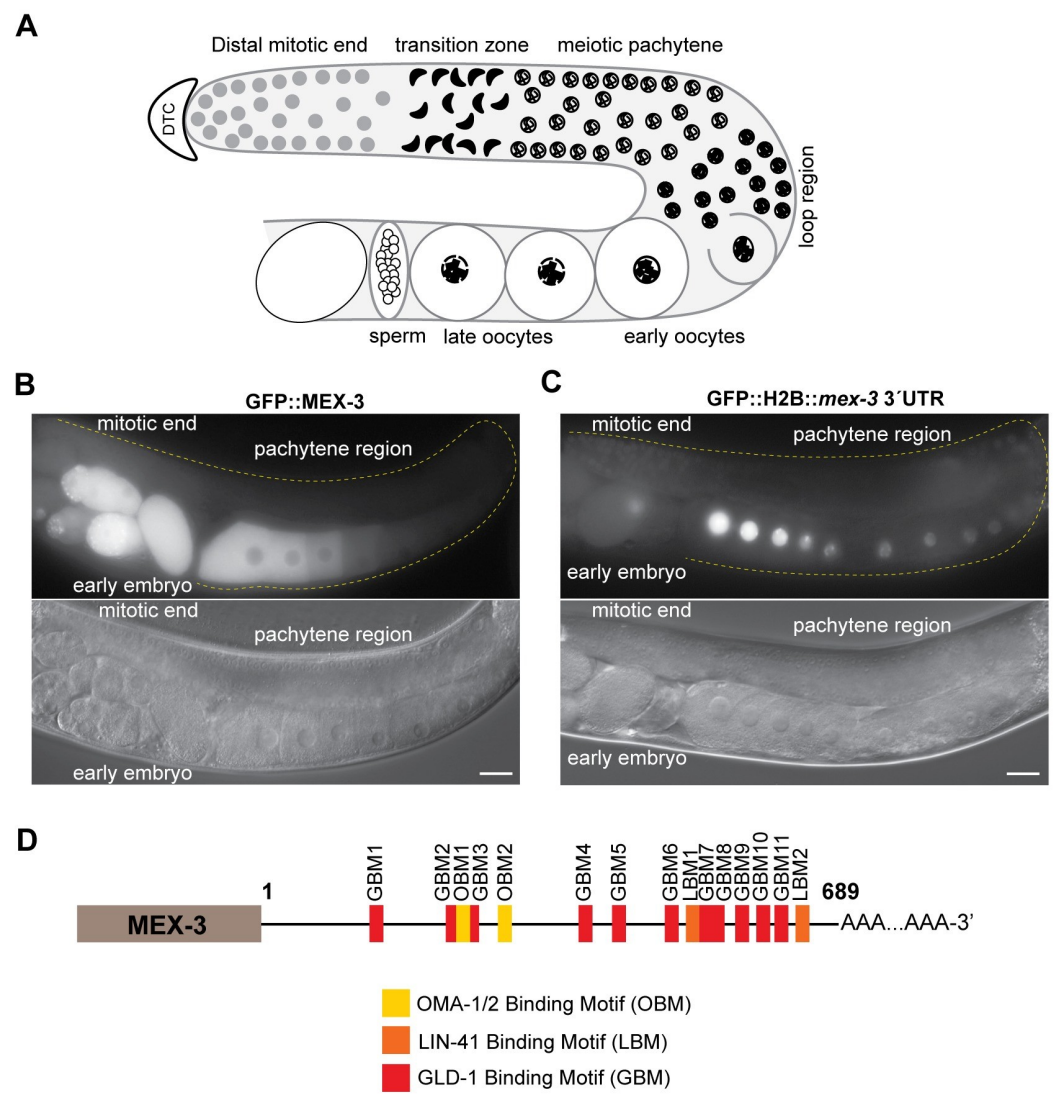
MEX-3 exhibits a unique expression pattern in the germline. **(A)** A schematic representing germline organization in *C*. *elegans*. One of two gonadal arms is represented. Germ cells undergo mitotic divisions in the distal end and then enter meiosis as they move farther from the distal tip cell. The syncytial meiotic nuclei start to recellularize around the loop region to form oocytes. In the proximal end, late oocytes undergo maturation, get fertilized by the sperm, and then move to the uterus to undergo embryonic development. **(B)** DIC and fluorescence images of an adult hermaphrodite germline from the strain in which MEX-3 is endogenously tagged with GFP (GFP::MEX-3) [38]. MEX-3 is present in the distal mitotic end, maturing oocytes, and early embryo. **(C)** DIC and fluorescence images of an adult hermaphrodite germline from the transgenic reporter strain carrying a pan-germline promoter fused to GFP and the *mex-3* 3´UTR [41]. The reporter is expressed in the distal mitotic end, maturing oocytes, and early embryo. **(D)** A schematic representing the 3´UTR of *mex-3* and its putative binding motifs. Images taken at 40x magnification. Scale bars = 30μm.

The germline of the hermaphroditic nematode *Caenorhabditis elegans* is a suitable model for studying spatiotemporal regulation of maternal mRNA [9,10]. The gonads consist of two symmetrical tube-shaped arms that contain mitotically dividing germ cells in the distal end of each tube (Fig 1A). As the mitotic nuclei move away from the distal end, they enter meiosis I and form a syncytium where the pachytene nuclei migrate to the periphery and share cytoplasmic content. The diplotene nuclei start to fully recellularize to form oocytes in the loop region as the tube bends. In the proximal end, oocytes undergo maturation and fertilization in the spermatheca where the sperm produced during the L4 larval stage or acquired by mating is stored. Embryogenesis continues in the uterus, where a hard chitin shell is secreted [11]. The 1-cell embryo undergoes multiple pre-determined cellular divisions to establish the body axes, segregate germline from soma, and define the number of tissue lineages before it exits the uterus [9].

UTR-mediated post-transcriptional regulation of maternal mRNAs is the major mechanism that controls gametogenesis and embryogenesis. Over the past few decades, dozens of germline RNA-binding proteins containing diverse and conserved RNA-binding domains have been identified and their biological functions characterized [12–17]. Several RBPs contribute to specific stages of germ cell development. For instance, the conserved maxi KH-domain RNA-binding protein GLD-1 promotes entry into meiosis in part by binding a specific regulatory element in the 3´UTR of the mitosis-promoting notch receptor *glp-1* and repressing its translation [18], the PUF-domain RNA-binding proteins FBF-1/2 promote mitosis in the distal end by preventing meiotic entry through 3´UTR-mediated translational repression of *gld-1* [19], and zinc finger RNA-binding proteins OMA-1/2 promote oocyte maturation in the proximal end through repression of multiple transcripts including *glp-1* [13,20–22].

A landmark paper by Seydoux and co-workers revealed that the 3´UTR is the primary determinant of patterned expression in the worm germline [23]. Germline promoters drive pan-germline expression, but the 3´UTR enables precise patterning within the germline. In an effort to match germline RBPs to their mRNA targets, the consensus binding motifs of several RBPs were defined by multiple groups using a variety of *in vitro* methods [12–14,16,17,24]. However, the motifs recognized by all worm RBPs investigated thus far are short, often six to eight nucleotides in length, and unlike microRNAs typically contain degenerate nucleotides. For the germline RBP GLD-1, the catalog of associated mRNAs in worms has also been defined using RIP-chip, PAR-CLIP, and HITS-CLIP [12,24–27]. These data validated and clarified the motif recognized by GLD-1 and provided a list of candidate target mRNAs. However, the congruence between data sets in site occupancy is modest, and in most cases, it remains unclear which binding events lead to a functional outcome. Similar data sets are only available for one other germline protein, the PUF-domain containing RNA-binding protein FBF-1 [28]. In most cases, the relative contribution of maternal 3´UTRs to reproductive fitness remains uncharacterized.

The highly conserved KH-domain RNA-binding protein MEX-3 promotes anterior cell fate specification in the embryo and contributes to maintenance of totipotency in the germline [29–31]. Null mutants of *mex-3* are maternal-effect embryonic lethal where the embryos fail to hatch due to cell fate patterning defects [31]. MEX-3 is evolutionarily conserved across multicellular animals. There are four human MEX-3 homologues (hMEX-3A-D) [32]. Some of these proteins function in cellular differentiation pathways [33,34]. For example, hMEX-3A regulates intestinal cell fate specification by 3´UTR-mediated negative regulation of the *cdx2* mRNA, which encodes a homeobox transcription factor [34]. The planarian homologue (mex3-1) maintains the pool of mitotic stem cells in addition to promoting stem cell differentiation [35]. In *Xenopus laevis*, mex3A contributes to maintenance of proliferating neuronal stem cells [36].

*In vitro* binding assays have shown that *C*. *elegans* MEX-3 binds two short motifs separated by zero to eight bases ((A/G/U)(G/U)AGN_(0–8)_U(U/A/C)UA) [16]. Each KH domain is predicted to bind one motif. MEX-3 is present in the distal mitotic end, maturing oocytes, and the early embryo where it also associates with P-granules [31,37,38], membrane-less structures composed of RNA and protein (Fig 1B and 1C). MEX-3 contributes to establishing the anterior/posterior asymmetry in the 1-cell embryo by repressing *pal-1* mRNA in the anterior blastomere and therefore restricting it to the posterior blastomere where PAL-1 is necessary for posterior cell fate specification [29,39]. MEX-3 also plays a role in maintaining totipotency in the germline; animals carrying null mutations in both *gld-1* and *mex-3* exhibit signs of trans-differentiation of the germ cells to neuronal or pharyngeal cells [30]. Previous studies have suggested that the RBPs GLD-1, LIN-41, and OMA-1/2 regulate MEX-3 expression. For GLD-1 and LIN-41, it is unknown whether this regulation is 3´UTR-mediated [38,40,41]. Additionally, the *mex-3* 3´UTR is sufficient to confer the MEX-3 pattern of expression to a reporter gene [23,41]. Transgenic animals carrying a reporter transgene driven by a pan-germline promoter fused to GFP and the 3´UTR of *mex-3* (*Ppie-1*::*GFP*::*H2B::mex-3 3*´*UTR*) exhibit an expression pattern that is similar to that of the endogenous MEX-3 (Fig 1B) [23]. Another transgenic strain carrying a different transgene (*Pmex-5*::*MODC PEST*::*GFP*::*H2B*:: *mex-3 3*´*UTR*, MODC: Mouse Ornithine DeCarboxylase) also shows a similar expression pattern (Fig 1C) [41]. While these studies demonstrate that the 3´UTR of *mex-3* is sufficient to pattern a reporter gene, it remained unknown whether the 3´UTR is necessary for the spatiotemporal expression of endogenous MEX-3 and whether it contributes to germline development and reproductive fitness.

In this study, we used CRISPR/Cas9 genome editing to demonstrate that the endogenous 3´UTR of *mex-3* is indeed essential for its spatiotemporal expression pattern in the germline. We further confirm that several RBPs regulate MEX-3 expression in different regions of the germline and show that this regulation is mediated through the 3´UTR. We also show that the 3´UTR is surprisingly dispensable for viability but does contribute to animal fecundity. Finally, we find that different regulatory mechanisms govern the expression pattern of MEX-3 in different regions of the germline, leading to its differential abundance across the gonad. Overall, our data demonstrate the importance of the 3´UTR to expression pattern and provide new insights into how RBPs control MEX-3 patterning.

## Results

### The 3´UTR of the endogenous *mex-3* locus is necessary for spatiotemporal expression

**Fig 2:**
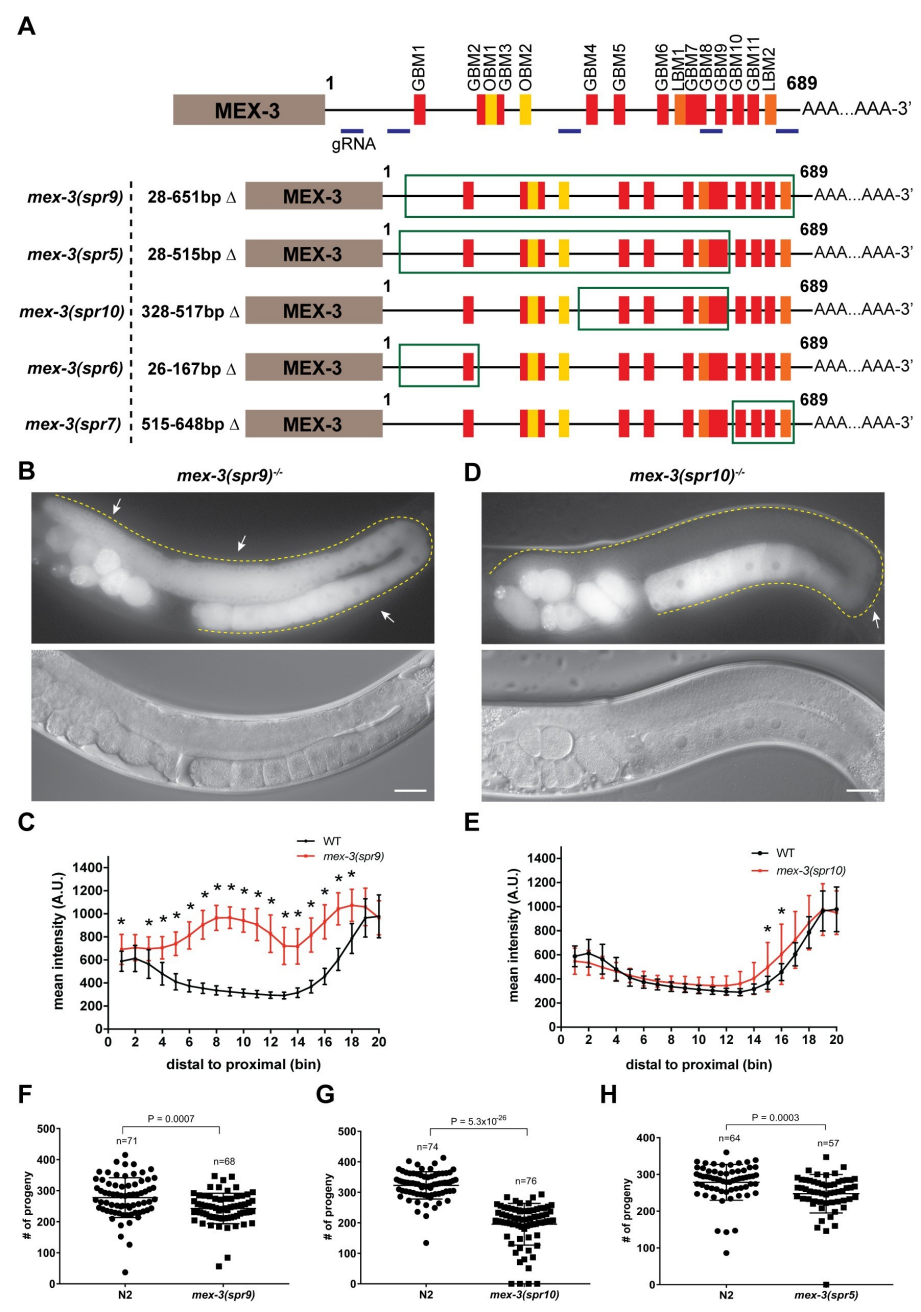
Deleting majority of the endogenous *mex-3* 3´UTR alters MEX-3 expression pattern and reduces fecundity. **(A)** A schematic representing the 3´UTR deletions made in the wild type GFP::MEX-3 strain. The region deleted in each mutant is highlighted by the green rectangle. The locations of the gRNAs used in CRISPR/Cas9 mutagenesis are highlighted. **(B)** DIC and fluorescence images of the germline of the homozygous *mex-3(spr9)* mutant animals. **(C)** quantitative analysis of the fluorescence intensity in the *mex-3(spr9)* mutant animals compared to that of wild type GFP::MEX-3 (n = 23). **(D)** DIC and fluorescence images of the germline of the homozygous *mex-3(spr10)* mutant animals. **(E)** quantitative analysis of the fluorescence intensity in the *mex-3(spr10)* mutant animals compared to that of wild type GFP::MEX-3 (n = 17). **(F)** brood size assay of homozygous *mex-3(spr9)* mutant animals at 20°C. Each dot represents the brood size of an individual animal. Data from three biological replicates are shown in the graph. P-values are from a Kolmogorov-Smirnov test. **(G)** brood size assay of homozygous *mex-3(spr10)* mutant animals at 20°C. **(H)** brood size assay of *mex-3(spr5)* mutant animals at 20°C. All p-values for panels C and E are reported in S4 Table. All images taken at 40x magnification. Scale bar = 30 μm.

Previous studies delineated the pattern of MEX-3 expression in the germline and embryos [29,31,37], and showed that the 3´UTR of *mex-3* is sufficient to confer that pattern to a transgene (Fig 1B and 1C) [23,41]. To investigate the biological consequence of regulation through the 3´UTR *in vivo*, we used CRISPR/Cas9 to make an allelic series of *mex-3* 3´UTR deletion mutants in a strain where the endogenous locus of *mex-3* is tagged with GFP (GFP::MEX-3) (Fig 2A) [38]. This background enables observation of changes in the expression pattern of GFP::MEX-3 in addition to scoring the resulting phenotypes. Initially, we wanted to test whether deleting majority of the 3´UTR or deleting smaller regions (100-200bp) would have an impact on MEX-3’s expression pattern. We reasoned that if bigger deletions did not alter the expression pattern, it is unlikely that small deletions that remove individual motifs would have an effect. We identified five potential guide RNA sites that are both efficient and unique to the *mex-3* locus (S3 Table). By microinjecting a mixture of Cas9 and two different guide RNAs, we were able to obtain several 3´UTR deletion mutants of various lengths including a mutant where majority of the 3´UTR was deleted. We generated five deletions (*spr6*: 142bp, *spr7*: 134bp, *spr10*: 190bp, *spr5*: 488bp, *spr9*: 624bp) in this series (Fig 2A). The *mex-3(spr9)* mutant animals contained the largest 3´UTR deletion (624bp out of 689 bp). Compared to wild type GFP::MEX-3, this mutant exhibited a significant increase in GFP::MEX-3 expression throughout the germline (Fig 2B and 2C, and S4 Table). The altered expression pattern of GFP::MEX-3 indicates that the endogenous 3´UTR is necessary for the spatiotemporal expression pattern of MEX-3 in the germline. The 3´UTR contains putative binding motifs for germline RBPs GLD-1, LIN-41, and OMA-1/2 (Figs 1D and S3). Previous studies indicated that all three RBPs contribute to the pattern of MEX-3 in the germline [38,40,41]. GLD-1 is expressed in the meiotic pachytene region where we observed de-repression of MEX-3 in this region in the *spr9* allele. LIN-41, an NHL-domain RBP, is expressed in the loop region where we also observed de-repression of MEX-3 in this allele [38]. OMA-1/2, zinc finger RBPs, are expressed in the oocytes and their expression increases as the oocyte approaches maturation [42]. We observed increased MEX-3 expression in all oocytes. Consistent with this work, multiple predicted binding motifs for each protein are removed in the deletion allele (S3 Fig). However, it is possible that regulation by additional RBPs, acting through yet-to-be-mapped cis-regulatory motifs, are also perturbed in the mutants, and it is not possible to assign specific changes in the pattern of expression to individual RBPs or motifs.

Among the shorter deletion mutants made in the GFP::MEX-3 background, only *mex-3(spr10)* mutant animals showed an increase of GFP::MEX-3, and this increase was modest and restricted to the loop region (Fig 2D and 2E, and S4 Table). This mutation resulted in a 190bp deletion that removed the 328-517bp region of the 3´UTR (Fig 2A). This region contains five putative binding motifs for GLD-1 (GBM) and one putative binding motif for LIN-41 (LBM: LIN-41 binding motif) (Fig 2A, 2D and 2E). GLD-1 protein starts to disappear in the loop region [43,44]. Thus, it is unlikely that this de-repression is due to the deletion of the GLD-1 GBMs. Additionally, *mex-3* was still repressed in the pachytene region where GLD-1 is highly expressed, indicating that the loss of the specific GLD-1 binding motifs in this mutant is not sufficient to de-repress MEX-3 in the pachytene region. Even when all five motifs were deleted, GFP::MEX-3 expression was not altered. Thus, our data suggest but do not prove that a different RBP, possibly LIN-41, contributes to repression of *mex-3* in the loop region.

**Fig 3:**
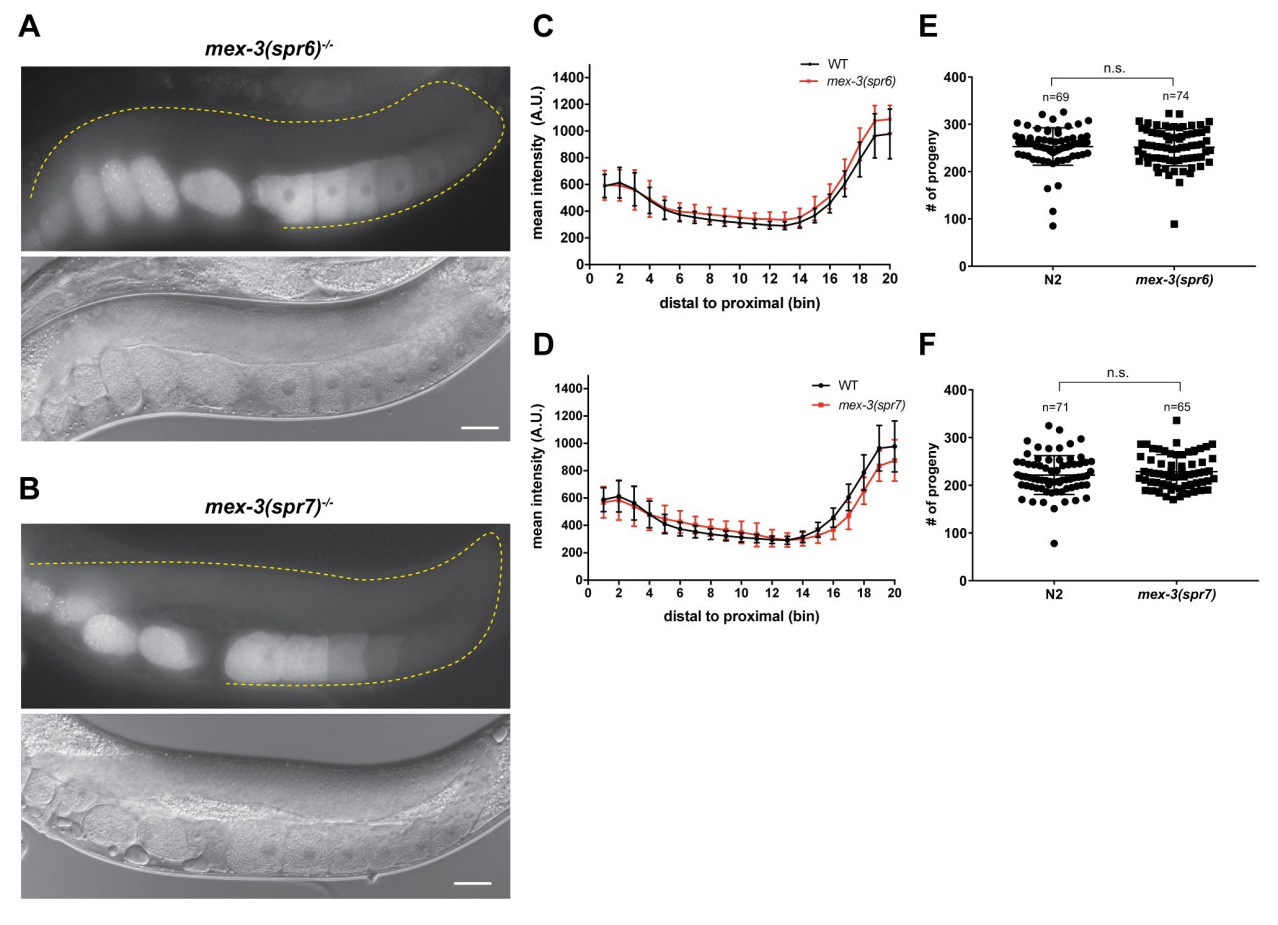
Deletion of the 26-167bp or 515-648bp regions of the *mex-3* 3´UTR does not alter MEX-3 expression pattern or affect fertility. **(A)** DIC and fluorescence images of the homozygous *mex-3(spr6)* mutant animals. **(B)** DIC and fluorescence images of the homozygous *mex-3(spr7)* mutant animals. **(C)** quantitative analysis of the fluorescence intensity in the homozygous *mex-3(spr6)* mutant animals compared to that of wild type GFP::MEX-3 (n = 16). **(D)** quantitative analysis of fluorescence intensity in the homozygous *mex-3(spr7)* mutant animals compared to that of wild type GFP::MEX-3 (n = 21). **(E)** brood size assay of homozygous *mex-3(spr6)* mutant animals at 20°C.There is no reduction in the brood size. **(F)** brood size assay of homozygous *mex-3(spr7)* mutant animals at 20°C. Each dot in panels E and F represents the brood size of an individual animal. In each panel, data from three biological replicates are shown in the graph. All p-values for panels C and D are reported in S4 Table. P-values in panels E and F are from a Kolmogorov-Smirnov test. All images taken at 40x magnification. Scale bar = 30 μm.

Neither *mex-3(spr6)* nor *mex-3(spr7)* mutant animals exhibited altered GFP::MEX-3 expression pattern in the germline (Fig 3A–3D). The mutation in the *mex-3 (spr6)* mutant animals removed another putative GLD-1 binding motif in the 3´UTR (Fig 2A). The lack of altered GFP::MEX-3 expression indicates that this putative motif is not necessary for repression of MEX-3. The mutation in the *mex-3(spr7)* removed three putative GLD-1 binding motifs and the second putative LIN-41 binding motif (Fig 2A). The lack of altered GFP::MEX-3 expression in this mutant indicates that none of these putative motifs are required to mediate repression of MEX-3 in the germline. Since *mex-3(spr5)* was made in a background strain in which MEX-3 is not tagged with GFP, we were not able to determine MEX-3 expression pattern as a result of the *spr5* deletion which removed the 28-515bp region of the 3´UTR (Fig 2A).

To assess whether any of the 3´UTR deletions disrupt poly(A) processing leading to aberrant 3´-end formation, we amplified and sequenced the 3´-end of polyadenylated *mex-3* transcripts produced by each mutant. None of the deletions affected the poly(A) processing site selection. All mutants appear to use the most common poly(A) processing site found in endogenous *mex-3* (S9 Table).

### The 3´UTR of *mex-3* is not required for viability but contributes to animal fecundity

All five mutants including the *spr9* allele that deletes majority of the 3´UTR are viable as homozygotes and can be easily propagated as such. The overall morphology appears normal in all variants. This demonstrates that the 3´UTR, though both necessary to establish the pattern of endogenous MEX-3 (this work) and sufficient to pattern reporter expression [23,41], is not essential for viability. To determine if the 3´UTR deletion mutations compromise reproductive health, we measured the brood size in homozygous mutant animals compared to control wild type animals. Three of the five deletion mutants exhibited reduced fecundity. Brood size was reduced in *mex-3(spr9)* (Fig 2F, p-value = 0.0007), *mex-3(spr10)* (Fig 2G, p-value = 5.3*10^−26^), and *mex-3(spr5)* (Fig 2H, p-value = 0.0003). Brood size was not reduced in the *mex-3(spr6)* and *mex-3(spr7)* mutant animals where MEX-3 expression was not altered (Fig 3E and 3F). While we do not know the MEX-3 expression pattern in the *mex-3(spr5)* mutant animals, these animals also exhibited reduced fecundity. This observation indicates that the 28-515bp region of the 3´UTR contributes to fertility. Intriguingly, the *mex-3(spr10)* mutant, which exhibited a modest increase of MEX-3 expression in the loop region, caused a reduction in brood size similar to that observed in the largest UTR deletion mutant *mex-3(spr9)*. These results suggest that deregulation of MEX-3 expression in the loop region of the germline may be sufficient to cause reduced fecundity. It is possible that de-repression of MEX-3 may alter gene expression in the loop region and influence oocyte formation, thus leading to a reduced brood size, but the exact mechanism remains unclear. Together, our data show that de-repression of MEX-3 does not cause sterility but does lead to reduced fecundity. These results also suggest that limiting MEX-3 expression pattern in the loop region may be necessary for optimal fertility.

**Fig 4:**
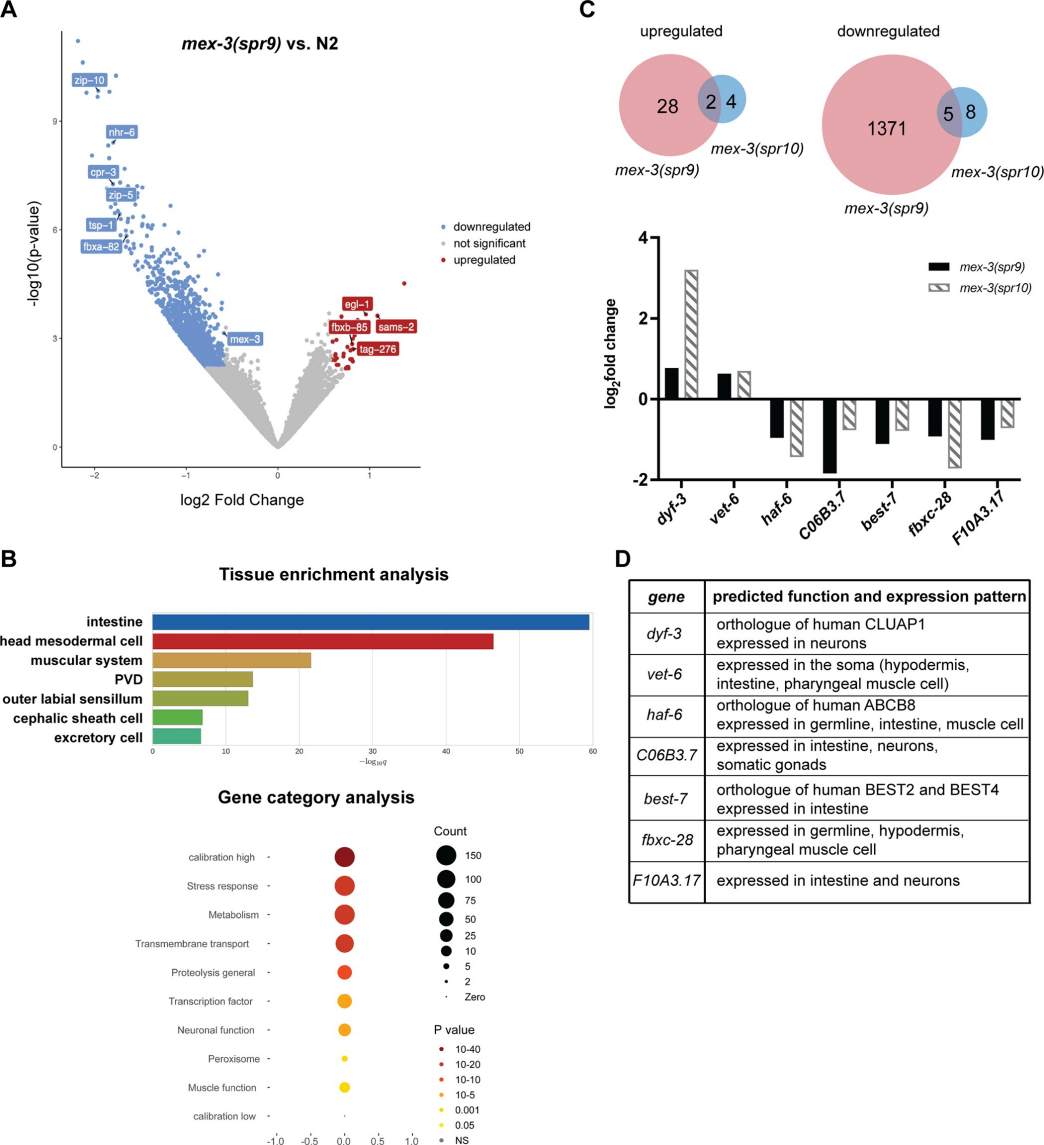
*mex-3* 3´UTR deletions in *mex-3(spr9)* and *mex-3(spr10)* mutants promote expression of somatic genes. **(A)** volcano plot of genes expressed in the *mex-3(spr9)* mutant animal. Differentially expressed genes are highlighted in blue (downregulated) or red (upregulated). **(B)** Tissue enrichment analysis and gene category analysis of the genes differentially expressed in *mex-3(spr9)* compared to N2. **(C)** Top: Venn diagrams showing the overlap in the upregulated and downregulated genes in *mex-3(spr9)* and *mex-3(spr10)* mutant animals. Bottom: plotted log2 Fold changes of the 7 overlapping genes that are differentially expressed in both *mex-3(spr9)* and *mex-3(spr10)* mutant animals. **(D)** Description of the predicted function and expression pattern of the genes in the graph in panel C.

To gain insight into how the *mex-3* 3´UTR deletions lead to altered MEX-3 expression pattern and reduced fecundity, we used RNA-seq to determine how the 3´UTR deletions affected *mex-3* mRNA levels and determine which genes are differentially expressed as a result (Fig 4). *mex-3* mRNA abundance was not altered in the *mex-3(spr10)* mutant animals, but it was significantly reduced in the *mex-3(spr9)* mutant animals (greater than 1.5-fold, p-value < 0.05) (Fig 4A). As such, *mex-3(spr9)* mutant animals exhibit increased MEX-3 protein expression throughout the germline but decreased *mex-3* mRNA levels. By contrast, *mex-3(spr10)* mutant animals display a modest increase in protein levels, but no change in *mex-3* mRNA levels. These results indicate that the increase of MEX-3 protein in both mutants is not caused by a similar increase in *mex-3* mRNA levels. Because both alleles mutate the 3´UTR, it is likely that the increase is caused by dysregulation of post-transcriptional mechanisms, although we cannot rule out other possibilities.

Differential gene expression analysis showed that 1406 genes were differentially expressed in *mex-3(spr9)* mutant animals compared to wild type N2 animals (Fig 4A). Thirty genes were upregulated in the mutant, while 1376 genes were downregulated (log_2_ fold change > 0.585, FDR < 0.05). Tissue enrichment analysis of these differentially expressed genes showed that they are mainly somatic genes expressed in tissues including the intestine, muscle, and neurons (Fig 4B). Both downregulated as well as upregulated genes showed this enrichment in somatic genes. On the other hand, only nineteen genes were differentially expressed in the *mex-3(spr10)* mutant (Fig 4C and S11 Table). Of these, seven genes were also differentially expressed in the *mex-3(spr9)* mutant. *dyf-3* and *vet-6* are upregulated in both mutants while *haf-6*, *C06B3*.*7*, *best-7*, *fbxc-28*, and *F10A3*.*17* are downregulated in both mutants (Fig 4C and 4D). All seven genes are normally expressed in somatic tissues except *haf-6* and *fbxc-28* which are expressed in the germline as well as the soma [45–47]. To determine whether the 3´UTRs of these genes contain MEX-3 recognition elements (MREs), we used a previously published method to predict putative MRE-containing UTRs [16]. *vet-6* and *best-7* each contain one MRE motif that is a perfect match to the MRE consensus sequence. All seven genes contain several putative MREs that are an imperfect match to the consensus MRE (S5 Fig). These results suggest that these genes could be direct targets of MEX-3. Surprisingly, *vet-6* contains a perfect MRE match in its 3´UTR but is upregulated in both *mex-3(spr9)* and *mex-3(spr10)* mutants. MEX-3 has only been shown to repress gene expression in its well-studied targets [16]. Nevertheless, we cannot disregard the possibility that *vet-6* is a direct target of MEX-3. It remains possible that MEX-3 could be a positive regulator in some contexts, as has been shown for other RBPs [48–51].

In *mex-3(spr9)*, the reduced fecundity phenotype could result from increased expression of specific somatic genes in the germline, reduced expression of others, or a combination of both. Gene category analysis of these differentially expressed genes showed enrichment in stress response, metabolism, and proteolysis pathways (Fig 4B). It could be that overexpression of MEX-3 causes downregulation of genes that mitigate stress, thus indirectly reducing fertility. In *mex-3(spr10)*, we predict that dysregulation of one or more of the nineteen differentially expressed genes underlies the reduced fecundity phenotype, possibly through misexpression in the loop region, where meiotic nuclei start to fully cellularize, leading to defects in oocyte cellularization or development. These hypotheses remain to be tested.

Together, our RNA-seq data show that *mex-3* mRNA levels are not precisely coupled to MEX-3 protein levels and suggest that multiple mechanisms may play a role in coordinating *mex-3* mRNA and protein abundance. Our data also show that overexpression of MEX-3 mainly affects the expression of somatic genes as opposed to germline-specific genes, consistent with its known role as a regulator of somatic gene expression programs in the embryo [29,31,39], and the increase in somatic tissue transdifferentiation upon loss of *mex-3* and *gld-1* in the germline [30].

### GLD-1, LIN-41, and OMA-1/2, but not DAZ-1 regulate *mex-3* expression in the germline through the 3´UTR

MEX-3 exhibits a unique expression pattern in the germline (Fig 1B and 1C) [31,37,38]. It was previously demonstrated that the 3´UTR is sufficient to confer the MEX-3 pattern of expression to a reporter gene in live animals (Fig 1C) [23,41]. Transcripts encoding *mex-3* have a long 3´UTR (689bp) that contains numerous putative binding motifs for several germline RBPs (Fig 1D). Thus, we predicted that the germline RBPs that associate with these motifs might coordinate MEX-3 expression through its 3´UTR. Previous findings suggested that GLD-1, LIN-41, and OMA-1/2 regulate MEX-3 expression [13,38,40,41]. However, we do not know whether GLD-1 and LIN-41 mediate *mex-3* expression through the 3´UTR. Thus, we used RNAi to first confirm the previous findings and then determine whether the regulation is 3´UTR-mediated. We soaked L4 or arrested L1 larval stage animals in dsRNA corresponding to the coding sequence of each candidate RBP. Then, we placed the animals on *E*. *coli* OP50 as a food source and imaged the adults using fluorescence microscopy to assess the effect of the RNAi on the germline GFP::MEX-3 expression pattern. We found that knockdown of *gld-1*, *lin-41*, or *oma-1/2* significantly altered the pattern of GFP::MEX-3 expression (S4 Fig and S6 Table), confirming prior results. In addition to these three RBPs, we also wanted to determine whether MEX-3 expression is regulated in the distal mitotic end. Thus, we also knocked down *daz-1*. DAZ-1 is an RNA Recognition Motif (RRM)-containing germline RNA-binding protein that is mainly expressed in the distal mitotic [52]. Surprisingly, we found that knockdown of *daz-1* resulted in significantly increased GFP::MEX-3 in the distal mitotic region (S4 Fig). Knockdown of *daz-1* results in defective oogenesis, so the impact on GFP::MEX-3 in the oocytes could not be directly assessed. DAZ-1 appears to be a novel regulator of MEX-3 expression in the distal mitotic end.

We also knocked down *daz-1*, *gld-1*, *lin-41*, or *oma-1/2* in the transgenic reporter strain described above where a pan-germline promoter (*Pmex-5*) drives the expression of a nuclear MODC PEST::GFP::*H2B* reporter under the control of the *mex-3* 3´UTR. Knockdown of *daz-1* did not show a strong increase of reporter expression in the distal mitotic end, suggesting that this protein alters MEX-3 expression via a 3´UTR-independent mechanism, possibly through the coding sequence, the 5´end, or indirectly through dysregulation of factors that act on the *mex-3* promoter.

**Fig 5:**
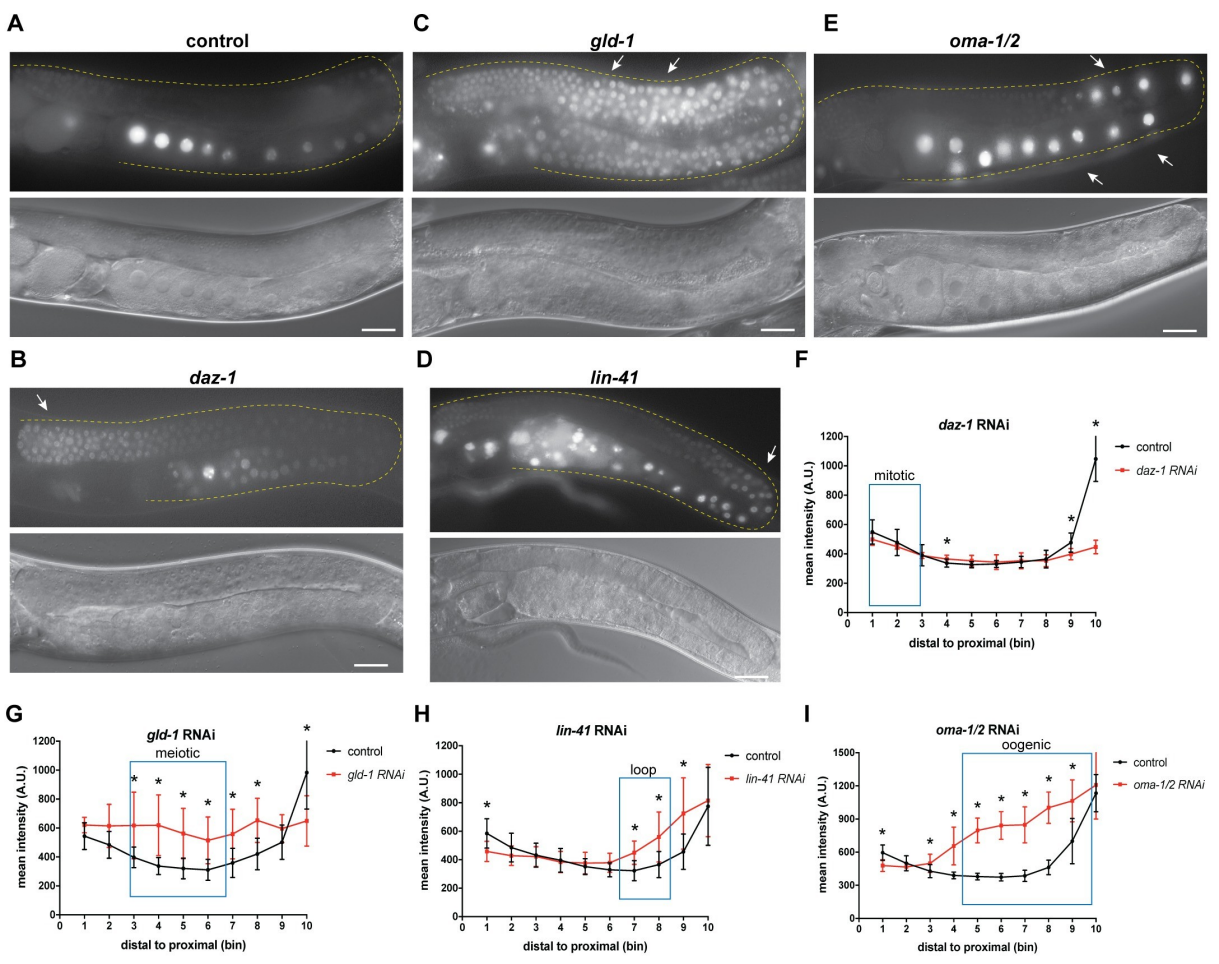
GLD-1, OMA-1/2, and LIN-41, but not DAZ-1 regulate spatiotemporal expression pattern of MEX-3 in the germline through the 3´UTR. **(A)** DIC and fluorescence images of *Pmex-5*::*MODC PEST*::*GFP*::*H2B*::*mex-3 3´UTR* animals from the control RNAi. **(B)** DIC and fluorescence images of *Pmex-5*::*MODC PEST*::*GFP*::*H2B*::*mex-3 3´UTR* animals after *daz-1* knockdown. *daz-1* knockdown didn´t cause increased reporter expression in the distal mitotic end. **(C)** DIC and fluorescence images of the transgenic reporter after *gld-1* knockdown. *gld-1* knockdown caused an overall increased expression of the reporter. **(D)** DIC and fluorescence of the transgenic reporter after *lin-41* knockdown. *lin-41* knockdown caused increased reporter expression in the loop region. **(E)** DIC and fluorescence images of transgenic reporter after *oma-1/2* knockdown. *oma-1/2* knockdown caused increased reporter expression in the oocytes [41]. **(F)** quantitative analysis of the reporter fluorescence intensity after *daz-1* knockdown (n = 10/10). **(G)** quantitative analysis of the reporter fluorescence intensity after *gld-1* knockdown (n = 8/8). **(H)** quantitative analysis of the reporter fluorescence intensity after *lin-41* knockdown (n = 8/8). **(I)** quantitative analysis of the reporter fluorescence intensity after *oma-1/2* knockdown (n = 14/14). (*) indicates statistical significance, p-value ≤ 0.05. All p-values for this figure are reported in S5 Table. Scale bar = 30 μm.

GLD-1 regulates various aspects of germline development [53,54]. Knockdown of *gld-1* resulted in accumulation of small oocytes, a phenotype characteristic of *gld-1* that corresponds to partial loss of function. Thus, the animals were able to undergo early stages of meiosis. Knockdown of *gld-1* resulted in de-repression of both endogenous MEX-3, which confirmed prior results, and the transgenic reporter in the meiotic pachytene region (Figs 5 and S4). Given that the 3´UTR of *mex-3* contains numerous putative GLD-1 binding motifs (Fig 1D), it is likely that GLD-1 represses *mex-3* expression in the meiotic region by directly binding some of these motifs in the 3´UTR. The observed de-repression of MEX-3 in the meiotic region in the *mex-3(spr9)* mutant allele is consistent with this possibility. Additionally, previous PAR-CLIP studies showed that *mex-3* is a direct target of GLD-1 [25]. However, we still do not know which GBMs in the 3’UTR are specifically utilized by GLD-1.

Knockdown of *lin-41* results in defects in the oogenic region where the oocytes are made but they are small and have abnormal morphology [55,56]. Knockdown of *lin-41* resulted in expansion of endogenous MEX-3, which confirmed prior findings, and the transgenic reporter to the loop region and late meiotic pachytene (Figs 5D and 5H and S4, and S5 Table), suggesting that LIN-41 may repress MEX-3 expression in the loop region through its 3´UTR. This is consistent with the observed de-repression of GFP::MEX-3 in loop region in *mex-3(spr9)* and *mex-3(spr10)* mutant alleles, both of which delete at least one of two putative LIN-41 motifs. Knockdown of *oma-1/2* results in formation of large defective oocytes that fail to complete oocyte maturation [42]. Knockdown of *oma-1/2* also resulted in increased expression of the transgenic reporter in the oocytes in the proximal end (Fig 5E and 5I). The 3´UTR of *mex-3* contains several clusters of UA(U/A) motifs predicted to bind to OMA-1/2 [13]. The *mex-3(spr9)* mutant allele showed increased MEX-3 abundance in all oocytes (Fig 2B and 2C). Previous tandem affinity purification studies demonstrated that *mex-3* transcripts associate with both LIN-41 and OMA-1, suggesting a direct interaction [38]. Together, our data add further support to the model that GLD-1, LIN-41, and OMA-1/2 directly regulate *mex-3* expression [38,40,41] and suggest that these RBPs act through the *mex-3* 3´UTR.

### Poly(A) tail length control mediates the spatiotemporal expression pattern of MEX-3

**Fig 6:**
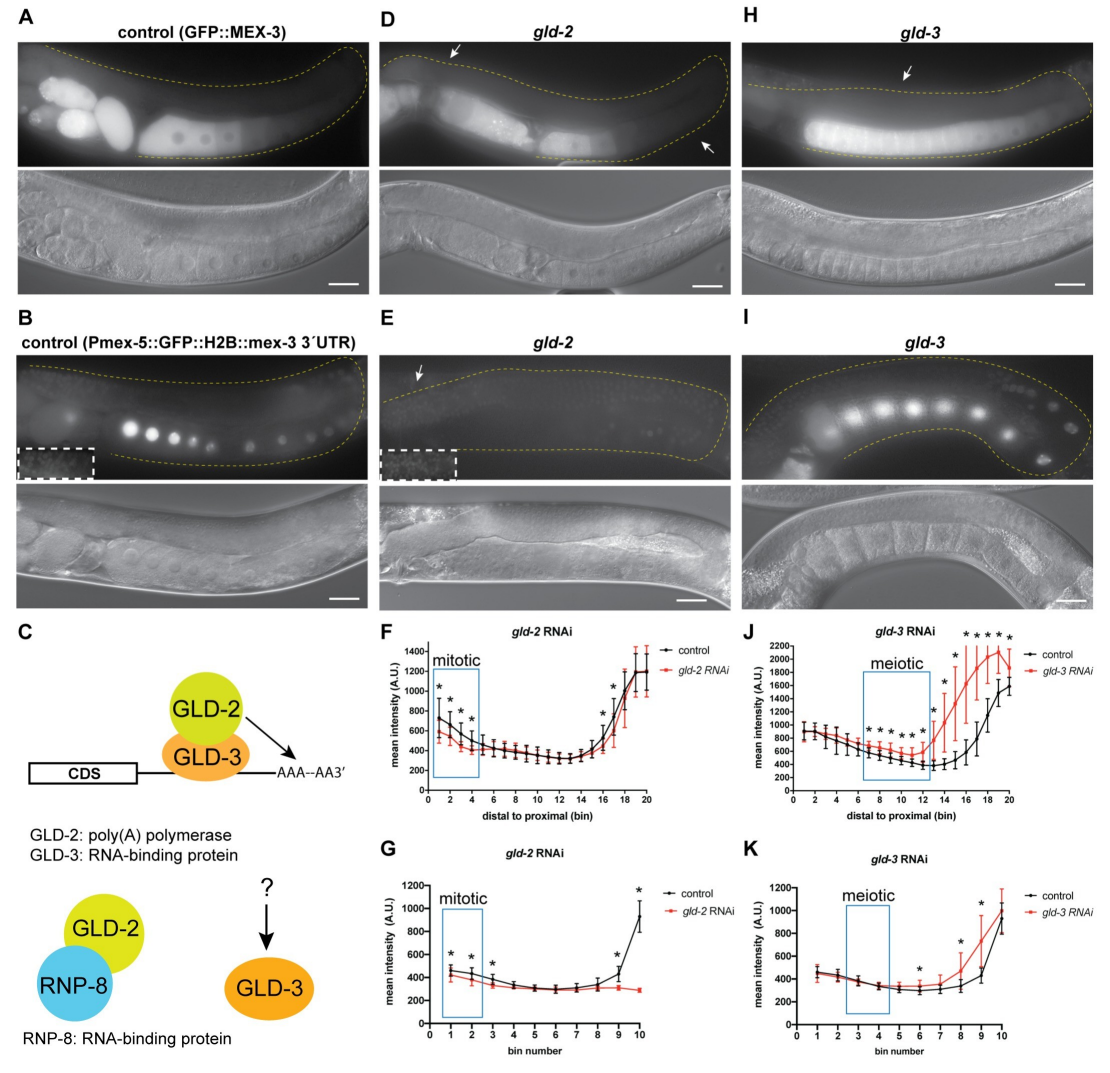
Polyadenylation contributes to regulation of MEX-3 in the germline. **(A)** DIC and fluorescence images of wild type GFP::MEX-3 animals from the control RNAi. **(B)** DIC and fluorescence images of the *gfp*::*mex-3 3*´*UTR* transgenic reporter strain from the control RNAi. **(C)** a model representing the known mechanism of GLD-2 and GLD-3 mediated polyadenylation. GLD-2, which lacks an RNA-binding domain, is thought to utilize the RBPs GLD-3 or RNP-8 in order to bind its target mRNAs. GLD-3 also exhibits functions independent of its role with GLD-2. **(D)** DIC and fluorescence images after *gld-2* knockdown. GFP::MEX-3 was significantly reduced in the distal mitotic end. **(E)** DIC and fluorescence images after *gld-2* knockdown in the transgenic reporter strain. Transgene expression was significantly reduced in the distal mitotic end. **(F)** quantitative analysis of fluorescence intensity after *gld-2* knockdown in the GFP::MEX-3 strain (n = 15/15). **(G)** quantitative analysis of fluorescence intensity after *gld-2* knockdown in the *gfp*:*mex-3 3*´*UTR* strain (n = 14/14). **(H)** DIC and fluorescence images after *gld-3* knockdown in the GFP::MEX-3 strain. GFP::MEX-3 expression was increased in the pachytene and oogenic regions. **(I)** DIC and fluorescence images after *gld-3* knockdown in the *gfp*::*mex-3* 3´UTR transgenic reporter strain. Transgene expression was not increased in the meiotic region but was increased in the oogenic region. **(J)** quantitative analysis of fluorescence intensity after *gld-3* knockdown in the GFP::MEX-3 strain (n = 7/12). **(K)** quantitative analysis of fluorescence intensity after *gld-3* knockdown in the *gfp*:*mex-3 3*´*UTR* strain (n = 10/10). (*) indicates statistical significance, adjusted p-value ≤ 0.05. All p-values for this figure are reported in S7 and S8 Tables. Scale bar = 30 μm.

The length of the poly-adenosine tail contributes to the stability and translational efficiency of eukaryotic mRNAs and has been demonstrated to control post-transcriptional regulation of maternal mRNAs in several metazoans including *C*. *elegans* [4,57,58]. To test whether cytoplasmic polyadenylation contributes to the pattern of MEX-3 expression, we used RNAi to knock down components of the germline cytoplasmic poly(A) polymerase complexes (*gld-2*, *gld-4*, *gld-3*, *rnp-8*) in the wild type GFP::MEX-3 strain (Fig 6 and S7 Table) and the *mex-3* transgenic reporter strain. GLD-2 and GLD-4 are the two main cytoplasmic poly(A) polymerases [49,59,60]. GLD-2 is a polymerase β nucleotidyltransferase. Unlike canonical nucleotidyltransferases, GLD-2 lacks an RNA-binding domain and thus requires an RBP co-factor [49]. Such factors include the bicaudal-C KH-domain RBP GLD-3 and the RRM-containing RBP RNP-8 (Fig 6C) [61,62]. GLD-2 is expressed throughout the entire germline. GLD-2 is required for the mitosis to meiosis transition as well as proper progression through early stages of meiosis [63]. Knockdown of *gld-2* resulted in defects in oogenesis where the meiotic nuclei progress through early stages of meiosis but fail to form normal oocytes and instead form defective oocytes [63]. Given that RNAi is partially penetrant, treated animals typically produced one or two oocytes. Knockdown of *gld-2* resulted in reduced GFP::MEX-3 expression in the distal mitotic end (Fig 6D and 6F, and S7 and S8 Tables). These results indicate that GLD-2 contributes to the positive regulation of MEX-3 in the distal mitotic end. Interestingly, we observed a similar phenotype when we knocked down *gld-2* in the transgenic reporter strain (Figs 6E and 5G, and S6 Table). This observation suggests that GLD-2 positively regulates MEX-3 expression in the distal mitotic end through its 3´UTR. Additionally, we observed a reduction in the GFP::MEX-3 expression as well as the transgene expression in the proximal region of the germline where defective oogenic cells were present. Since *gld-2* RNAi prevents meiotic nuclei from completing pachytene and completing oocyte formation [63], we cannot make a clear conclusion about the role for GLD-2 in *mex-3* regulation in the oocytes.

GLD-3 is required for the mitosis to meiosis switch as well as the spermatogenesis to oogenesis switch in hermaphrodites [61,64]. Knockdown of *gld-3* results in defective spermatogenesis and thus leads to oocyte arrest and accumulation in the proximal region of the germline. Previous studies have shown that oocyte arrest leads to formation of RNP foci in the oocytes and that MEX-3 is a component of these foci [37,65]. Confirming these prior observations, GFP::MEX-3 accumulated in punctate-like formations surrounding the nucleus and near the plasma membrane (Fig 6H). Interestingly, we also observed increased GFP::MEX-3 expression in the pachytene region indicating that GLD-3 may be involved in negative regulation of MEX-3 in that region (Fig 6H and 6J, and S7 Table), although it remains unknown whether this regulation is direct or indirect. We did not observe a similar increase in the *mex-3* 3´UTR reporter strain which indicates that this effect is not 3´UTR mediated (Fig 6I and 6K, and S8 Table). It is possible that GLD-3 negatively regulates MEX-3 through an unknown mechanism or that GLD-3 positively regulates a negative regulator of MEX-3 expression in the pachytene region. Neither knockdown of *gld-4* nor *rnp-8* changed the expression of GFP::MEX-3. Together, these data suggest that GLD-2 and GLD-3 contribute to regulating wild type GFP::MEX-3 expression in the germline. GLD-2 appears to positively regulate expression of MEX-3 in the distal mitotic end while GLD-3 appears to be an indirect negative regulator of MEX-3 expression in the meiotic pachytene region. GLD-2 and GLD-3 are likely acting through separate pathways since they also exhibit functions that are independent of each other [62,64,66].

**Fig 7:**
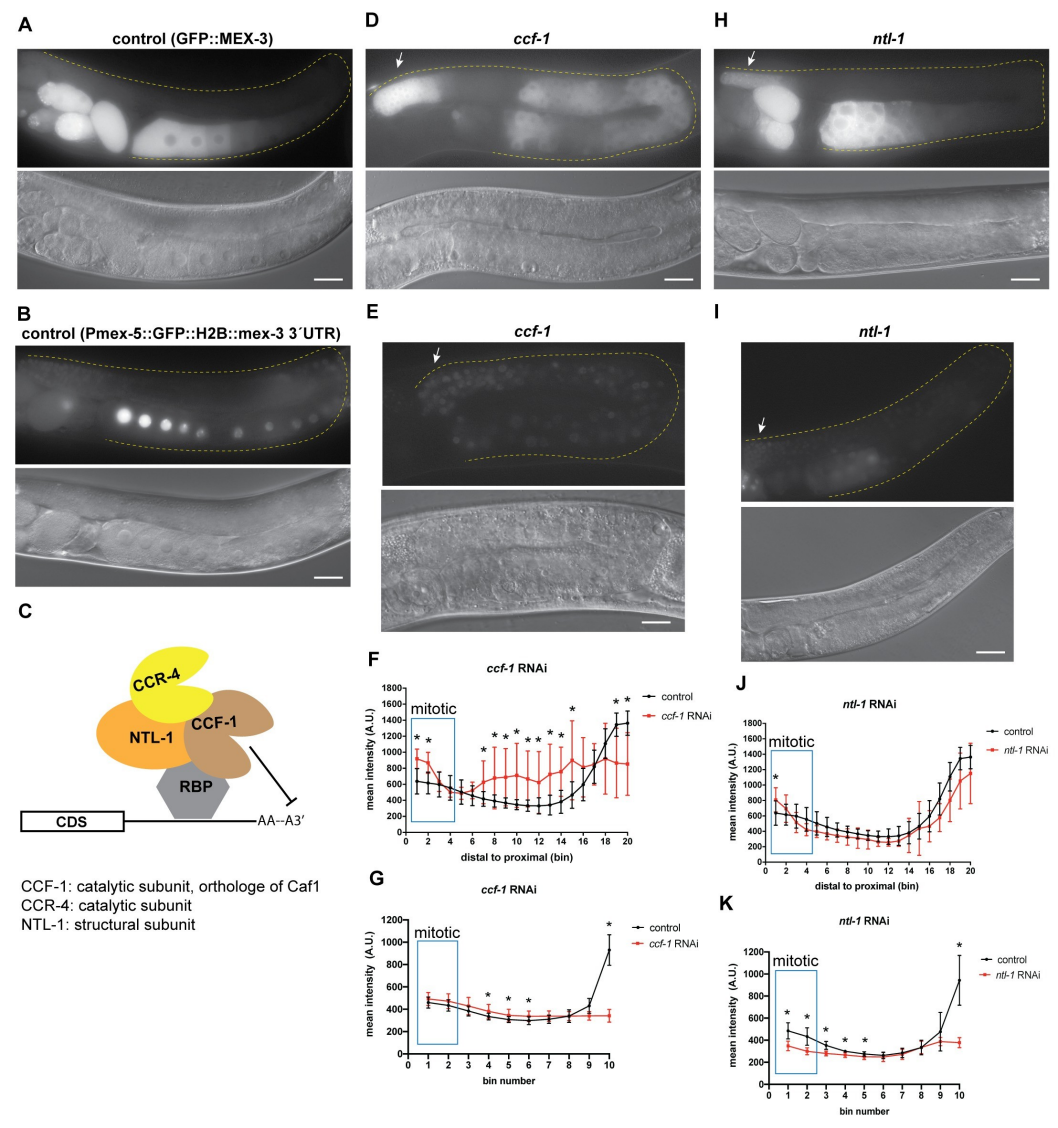
De-adenylation contributes to regulation of MEX-3 in the germline. **(A)** DIC and fluorescence images of wild type GFP::MEX-3 animals from the control RNAi. **(B)** DIC and fluorescence images of the *gfp*::*mex-3 3´UTR* transgenic reporter strain from the control RNAi. **(C)** a model representing the known mechanism of CCF-1, NTL-1, and CCR-4 mediating poly(A) deadenylation. CCF-1 and CCR-4 are the deadenylases while NTL-1 is a structural subunit. CCF-1 is the major de-adenylase. All three proteins are thought to form a complex. **(D)** DIC and fluorescence images after *ccf-1* knockdown in the GFP::MEX-3 strain. GFP::MEX-3 expression was significantly increased in the distal mitotic end. **(E)** DIC and fluorescence images after *ccf-1* knockdown in the *gfp*::*mex-3 3´UTR* transgenic reporter strain. **(F)** quantitative analysis of fluorescence intensity after *ccf-1* knockdown in the GFP::MEX-3 strain (n = 15/15). **(G)** quantitative analysis of fluorescence intensity after *ccf-1* knockdown in the transgenic reporter strain (n = 12/12). **(H)** DIC and fluorescence images after *ntl-1* knockdown in the GFP::MEX-3 strain. GFP::MEX-3 expression was significantly increased in the distal mitotic end. **(I)** DIC and fluorescence images after *ntl-1* knockdown in the *gfp*::*mex-3 3´UTR* transgenic reporter strain. Transgene expression was not reduced in the distal mitotic end. **(J)** quantitative analysis of fluorescence intensity after *ntl-1* knockdown (n = 13/13) in the GFP::MEX-3. **(K)** quantitative analysis of fluorescence intensity after *ntl-1* knockdown (n = 13/13). (*) indicates statistical significance, adjusted p-value ≤ 0.05. All p-values for this figure are reported in S7 and S8 Tables. Scale bar = 30 μm.

In *C*. *elegans*, the major deadenylation complex consists of the subunits CCF-1, CCR-4, and NTL-1 (Fig 7C). CCF-1 is an orthologue of the highly conserved eukaryotic catalytic subunit CAF1 [67,68]. CCR-4 is an orthologue of the other conserved catalytic subunit CCR4. NTL-1 is an orthologue of the scaffolding protein NOT1. These proteins are expressed in both germline and somatic tissues. Biochemical analyses have shown that these proteins form a complex in *C*. *elegans*. Both CCF-1 and NTL-1 are essential for fertility while CCR-4 has a minor impact. CCF-1 is the major de-adenylase in the complex [68].

To assess the role of cytoplasmic deadenylation in regulating MEX-3, we knocked down these components using RNAi in the GFP::MEX-3 strain as well as the *mex-3* 3´UTR transgenic reporter strain. Knockdown of either *ccf-1* or *ntl-1* altered the expression pattern of GFP::MEX-3 (Fig 7). Knockdown of *ccf-1* causes meiotic defects and disorganization of the germline where multiple layers of oocytes appear in the proximal end. The oocytes are small and fail to complete oocyte maturation [67,68]. In wild type animals, meiotic nuclei continue to undergo prophase I in the early oocytes and then undergo prophase II in the most proximal end of the germline. Knockdown of *ccf-1* prevents these meiotic nuclei from completing prophase II and maturation. Thus, the oocytes we observed likely resemble early oocytes. Knockdown of *ccf-1* resulted in increased expression in the mitotic region and ectopic expression in the oocytes where some defective oocytes appeared to have varying levels of GFP::MEX-3 (Fig 7D and 7F, and S7 Table). These results suggest that CCF-1 negatively regulates *mex-3* expression in the distal mitotic end. Knockdown of *ntl-1* also causes defects in meiotic progression and leads to defects in germline organization in the proximal end of the germline where small defective oocytes appear in multiple layers in that region. Knockdown of *ntl-1* resulted in increased expression of GFP::MEX-3 in the mitotic region (Fig 7H and 7J, and S7 Table), suggesting NTL-1 contributes to negative regulation of MEX-3 in the distal mitotic end. It is not clear whether the increase in GFP::MEX-3 expression observed upon *ccf-1* or *ntl-1* knockdown is direct or indirect. It is possible that knockdown of these components increases the expression of a positive MEX-3 regulator in the distal mitotic end. We cannot make a conclusion about proximal regulation due to disorganization of this region of the germline upon knockdown of either factor.

Consistent with the indirect regulation hypothesis, knockdown of *ccf-1* in the *mex-3* 3´UTR transgenic reporter did not increase transgene expression in the mitotic region (Fig 7E and 7G, and S8 Table), indicating that CCF-1 does not mediate repression of *mex-3* through its 3´UTR in this region. Interestingly, in contrast to endogenous MEX-3, transgene expression appeared reduced in the distal mitotic end after knockdown of *ntl-1* in the *mex-3* 3´UTR transgenic reporter strain. This result suggests that NTL-1 may positively regulate MEX-3 expression through its 3’UTR when this regulatory circuit is isolated. It is also possible that this phenotype is indirect.

Knockdown of *ccr-4* does not alter meiotic progression or germline organization. Here, knockdown of *ccr-4* did not alter the GFP::MEX-3 expression suggesting that CCR-4 alone does not contribute to regulation of MEX-3 expression. Together, these results show that components of the deadenylation machinery repress GFP::MEX-3 expression in the mitotic region, but do not distinguish direct from indirect effects.

### Translation initiation factor IFE-3 regulates MEX-3 expression

**Fig 8:**
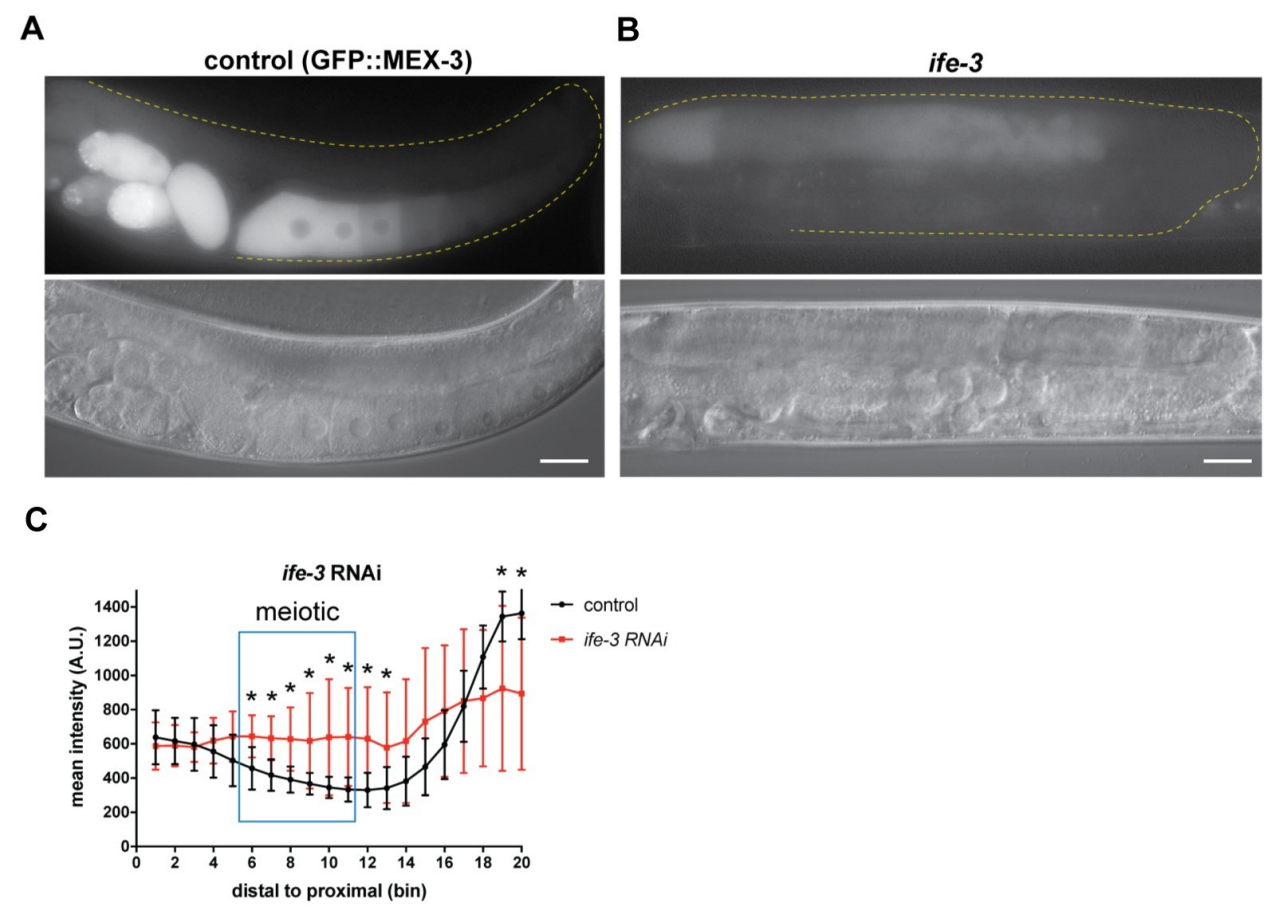
Translation initiation factor IFE-3 contributes to repression of MEX-3 in the meiotic pachytene region. **(A)** DIC and fluorescence images of wild type GFP::MEX-3 animals from the control RNAi. **(B)** DIC and fluorescence images after *ife-3* knockdown. GFP::MEX-3 expression was significantly increased in the meiotic region. **(C)** quantitative analysis of fluorescence intensity after *ife-3* knockdown (n = 13/13). (*) indicates statistical significance, adjusted p-value ≤ 0.05. All p-values for this figure are reported in S7 Table. Scale bar = 30 μm.

There are five *C*. *elegans* translation initiation factor eIF4E homologs. Among these factors, only *ife-3* causes embryonic lethality when knocked down alone under normal growth conditions [69]. A recent report revealed that this factor negatively regulates the translation of specific maternal transcripts in the germline, presumably by interfering with normal translation initiation mediated by the other homologs [70]. To test whether *ife-3* contributes to the pattern of MEX-3 expression, we used RNAi to knock it down in the GFP::MEX-3 strain. Knockdown of *ife-3* caused defects in late stages of meiosis leading to the failure to form oocytes (Fig 8 and S7 Table). We observed an increase of GFP::MEX-3 levels in the meiotic region. These results suggest that IFE-3 negatively regulates expression of MEX-3 in the meiotic region, but it remains unknown whether this regulation is direct or indirect. The disorganization of the proximal germline precludes interpretation of this region in *ife-3* knockdown.

## Discussion

Previous studies have shown that the 3´UTR is a major contributor to reporter gene expression in the germline [23]. However, the biological role of the 3´UTR of endogenous germline genes, including those encoding RNA-binding proteins with key regulatory functions, has not been directly tested. Defining the importance of the endogenous germline 3´UTRs to germline development and gene expression is key to understanding the overall importance of maternal RNA regulation. Our results show that the endogenous 3´UTR is necessary for repression in the germline, and that full repression requires several RBPs, control of the poly(A) tail length, and inhibition by a translation initiation factor. Our results also show that the 3´UTR of *mex-3* is surprisingly dispensable for embryonic viability but does contribute to overall fecundity. How these two phenotypes are linked remains to be determined.

Our results are consistent with and expand upon a previous study that showed GLD-1 represses *mex-3* expression in the germline [40]. The 3´UTR of *mex-3* is predicted to contain eleven putative GLD-1 binding motifs (GBMs) across its length [12,24]. Consistent with previous findings, our results reveal that *gld-1* knockdown leads to de-repression of the endogenous MEX-3 in the meiotic region. The observation of a similar result in the *mex-3* 3´UTR reporter transgenic strain indicates that this repression is 3´UTR-mediated. It is likely that redundancy exists between the multiple GBMs found throughout the UTR, and that de-repression requires deletion of all of them. Consistent with this hypothesis, *mex-3(spr9)* mutant animals, which lack the majority of the 3´UTR including all eleven predicted GBMs, showed complete de-repression of MEX-3 in the meiotic region (Fig 2B and 2C). Among the shorter 3´UTR deletion mutants, *mex-3(spr6)* removes a single GBM, *mex-3(spr7)* removes three, and *mex-3(spr10)* removes a different set of five GBMs (Fig 2A). None of these mutations caused increased MEX-3 levels in the meiotic region, suggesting that none of the GBMs within are necessary for repression in the presence of intact GBMs elsewhere in the UTR. Thus, the precise position of GLD-1 binding within the UTR is likely not important to repressive activity. Given that GLD-1 is expressed in the meiotic pachytene region and that none of the other RBPs with predicted binding motifs in the 3’UTR of *mex-3* are expressed in this region, we predict that the de-repression of MEX-3 in the meiotic region in the *mex-3(spr9)* mutant allele is likely due to deletion of GLD-1 binding motifs. Our RNAi results and previously published data [40] support this interpretation. However, we cannot rule out that regulation by additional RBPs acting through unmapped cis-regulatory motifs also contributes to repression. The precise contribution of GLD-1 to *mex-3* repression remains to be directly tested via specific mutations of the eleven predicted GBMs in the *mex-3* 3’UTR in various combinations.

**Fig 9:**
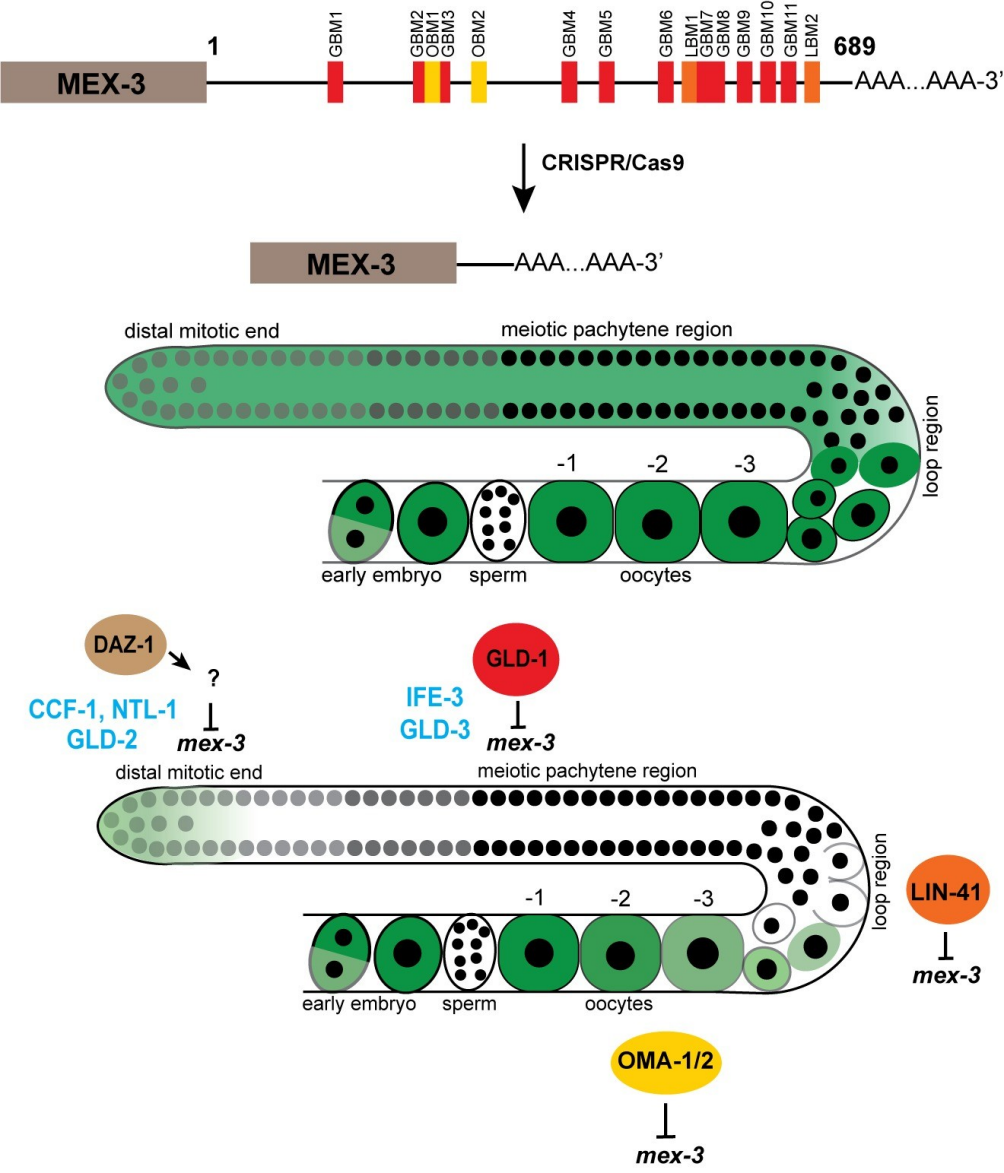
Model for 3´UTR-mediated post-transcriptional regulation of *mex-3* in the germline. The endogenous 3**´**UTR is required for the spatiotemporal expression pattern of MEX-3. Deletion of majority of the 3’UTR causes increased GFP expression throughout the germline. DAZ-1 indirectly regulates expression of MEX-3 in the distal mitotic end. GLD-1 represses MEX-3 expression through its 3´UTR in the meiotic pachytene region. LIN-41 represses expression of MEX-3 in the loop region while OMA-1/2 repress expression of MEX-3 in the maturing oocytes. Cytoplasmic polyadenylation positively regulates expression of MEX-3 in the distal mitotic end. Cytoplasmic deadenylation negatively regulates expression of MEX-3 in the distal mitotic end. IFE-3 negatively regulates expression of MEX-3 in the meiotic pachytene region.

GLD-1 may repress *mex-3* expression by inhibiting its translation [71]. Consistent with this hypothesis, knockdown of the translational repressor *ife-3* showed de-repression of MEX-3 in the meiotic pachytene region where GLD-1 mediates *mex-3* repression. IFE-3 and GLD-1 could work together to inhibit translation of *mex-3* in the meiotic region. However, it has been shown by others that the translational efficiency of *gld-1* is reduced after *ife-3* knockdown [70], suggesting a second possibility that IFE-3 may indirectly repress *mex-3* by positively regulating a negative *mex-3* regulatory RBP. Taken together, our findings support a model where GLD-1 represses *mex-3* translation through its 3´UTR (Fig 9). It remains to be determined how GLD-1 represses translation of *mex-3*.

GFP::MEX-3 expression was previously shown to expand to the loop region in a *lin-41* null mutant background [38]. Consistent with these findings, our results show that LIN-41 represses *mex-3* in the loop region through its 3´UTR, which was previously unknown. This is also consistent with a study that revealed that *mex-3* mRNA associates with purified recombinant LIN-41 in an *in vitro* pull-down assay [38]. The 3´UTR deletion in the *mex-3(spr10)* mutant animals that removes one putative LIN-41 binding motif (LBM) (Fig 2A), showed de-repression of MEX-3 in the loop region. In contrast, *mex-3(spr7)* mutant animals, which delete the other putative LBM, did not. These data suggest that the motif deleted in the *spr10* allele may be functional in *mex-3* repression, while that in *spr7* may not. Interestingly, *mex-3(spr9)* mutant animals showed stronger de-repression of MEX-3 in the loop region than *mex-3(spr10)* animals. This observation suggests that both LIN-41 motifs may be necessary for maximal repression of *mex-3* in the loop region, but this will need to be addressed through additional mutants that perturb both binding sites without affecting candidate motifs for other RBPs. Neither GLD-1 nor OMA-1/2 are expressed in the loop region. Thus, the de-repression observed in the loop region of the *mex-3(spr9)* and *mex-3(spr10)* mutant alleles is not likely to be due to repression mediated by these RBPs. The reduced fertility observed in the *mex-3(spr10)* mutant allele could be due to misregulation of gene expression in the loop region. Indeed, our RNA-seq data identify several potential candidates that could contribute to the phenotype. It is intriguing that the reduction in fecundity appears greater in the *mex-3(spr10)* allele compared to the *mex-3(spr9)* allele. The basis for this difference is unknown, but we hypothesize that differences in gene expression observed in the RNA-seq data may account for the apparent difference. Together, our findings show that the 3´UTR of *mex-3* is necessary for repression of MEX-3 in the loop region, and that at least one of two LIN-41 motifs is necessary for MEX-3 repression in this region.

OMA-1/2 appear to repress *mex-3* expression in the oocytes through the 3´UTR (Fig 5E and 5I, and S7 Table) [38,41]. Consistently, *mex-3* mRNA was found to associate with purified OMA-1 protein *in vitro* [38]. The 3´UTR of *mex-3* contains multiple clusters of putative OMA-1 binding motifs UA(A/U). We previously demonstrated that OMA-1 binds to UA(A/U)-enriched motifs with a high degree of cooperativity [13]. Only *mex-3(spr9)* mutant animals showed increased GFP::MEX-3 expression in the proximal oogenic region. It is possible that OMA-1/2 may bind UA(A/U)-enriched clusters in the 3´UTR of *mex-3* to repress its expression in the maturing oocytes, but this hypothesis remains to be directly tested through mutations that disrupt OMA-1/2 interactions without affecting the binding of other RBPs. Intriguingly, knockdown of *oma-1/2* caused a significant increase in the amount of GFP::MEX-3 in oocyte nuclei. This suggests that OMA-1/2 plays a role in MEX-3 partitioning between the nucleus and the cytoplasm in oogenesis. It remains unknown what role MEX-3 plays in the nucleus, if any, or if it actively shuttles between the two compartments.

DAZ-1, an RRM-containing RBP that contributes to meiotic progression during oocyte development [52], appears to regulate the expression pattern of MEX-3 in the mitotic region. Although MEX-3 is expressed in that region in wild type animals, knockdown of *daz-1* caused a significant increase of GFP::MEX-3 expression in the distal mitotic end. The binding specificity of DAZ-1 is unknown. Therefore, we do not know if the 3´UTR of *mex-3* contains binding motifs for DAZ-1. However, knockdown of *daz-1* in the *mex-3* 3´UTR reporter strain did not show similar results in the distal mitotic end. Although DAZ-1 may not regulate MEX-3 expression through its 3´UTR, it is possible that it may regulate the expression of other pathways that directly influence *mex-3* expression in that region. DAZ-1 was previously shown to positively regulate expression of *gld-1* through its 3´UTR [72]. Thus, it is possible that DAZ-1 positively regulates expression of a factor that negatively regulates MEX-3 expression in the distal mitotic end. Precisely how DAZ-1 contributes to this pattern, and whether it works through cytoplasmic deadenylation, is still unknown.

Our findings demonstrate that the 3´UTR of *mex-3* is necessary for the unique spatiotemporal expression pattern of MEX-3 in the germline (Fig 9) and contributes to germline development and fecundity. Surprisingly, *mex-3* mRNA levels were reduced in *mex-3(spr9)*, while MEX-3 protein was increased throughout the germline. We hypothesize that the 3´UTR contains both a negative regulatory circuit acting at the level of translation, and a positive regulatory circuit acting at the level of mRNA stability. While this hypothesis remains to be tested, our data reveal that multiple regulatory mechanisms are required to generate the expression pattern of endogenous *mex-3* mRNA and protein.

Overexpression of MEX-3 could lead to the observed reduced fecundity if increased MEX-3 concentration in the germline leads to greater occupancy of sub-optimal MEX-3 binding motifs on both existing and new target transcripts. This may cause dysregulation of expression of those genes in the germline, reducing but not eliminating gamete production. Some candidates, identified by RNA-seq of the mutants compared to wild type, are presented above. It is also possible that MEX-3 in the germline may undergo post-translational modifications that regulate its activity levels, such that abundance does not directly correlate to regulatory activity. In the early embryo, MEX-3 localizes to both the anterior and posterior blastomeres. However, MEX-3 is only active in the anterior blastomere due to degradation of MEX-3 in the posterior blastomere. In the anterior, the RBPs MEX-5/6 are thought to bind and protect MEX-3. The degradation in the posterior blastomere is mediated by the RBP SPN-4 and the kinase protein PAR-4 [29]. SPN-4 is only expressed in late oocytes and the early embryo [38,40,50], but PAR-4 is expressed throughout the germline and in the early embryo [73]. Therefore, PAR-4 could potentially mediate degradation of fractions of MEX-3 in the germline. Both post-transcriptional as well as post-translational regulatory mechanisms may contribute to MEX-3 expression pattern in the germline. However, we note that the 3´UTR is sufficient to pattern the expression of a reporter gene, so post-translational regulation through directed MEX-3 turnover may enforce the pattern of expression but is not absolutely required.

It will be intriguing to assess whether the endogenous 3´UTRs of other germline RBPs are equally dispensable for fertility. Most studies investigating germline RBPs function in *C*. *elegans* have relied on transgenic reporter strains [14,16,23,74–77]. By targeting the endogenous 3´UTRs using CRISPR/Cas9 genome editing, we can assess the importance of the UTR elements to biological function. This approach can also be applied in other organisms where key proteins exhibit unique spatial expression patterns to control early developmental processes. Another important direction is validation of the putative RBP binding motifs which were determined using *in vitro* binding assays. It will be important to use *in vivo* binding methods such as CLIP-based assays to investigate the endogenous binding specificity of many of these germline RNA-binding proteins and combine such studies with genetics and biochemical analyses to further dissect UTR-RBP interaction networks and functionality.

## Materials and methods

### Worm maintenance

All strains used were maintained by growing the animals on *E*. *coli* OP50 seeded NGM plates. N2 wild type strain was used as a control in all the brood size experiments. Each isolated mutant was outcrossed at least three times before analysis. Genotypes of all the strains in this paper are in S1 Table.

### RNAi

RNAi was performed by soaking animals in double-stranded RNA corresponding to the genomic cDNA sequence of the gene of interest. RNA was isolated from wild type N2 animals using trizol and phenol-chloroform extraction followed by RT-PCR using Superscript III One Step RT-PCR system with Platinum Taq DNA polymerase kit (ThermoFisher Scientific cat #: 12574026) to prepare the cDNA, which was used to amplify the template DNA used in the in vitro transcription (IVT) reaction to transcribe the dsRNA. Ambion MEGAscript T7 in vitro transcription kit (ThermoFisher Scientific cat #: AM1333) was used to prepare the dsRNA following the manufacturer´s protocol. The dsRNA was purified by phenol-chloroform extraction and isopropanol precipitation. The sequences of the oligos that were used to amplify the cDNA used as a template in the IVT reactions are in S2 Table. For the RNAi soaks, each tube contained 2μl of 5x soaking buffer, and 8μl of 500-1000ng/μl purified dsRNA. 0.5μl of M9 buffer containing arrested L1 animals was added to each individual tube. In the *lin-41* RNAi, L4 animals were placed in the dsRNA instead of L1s. The control tube contained 2μl of soaking buffer and 8μl of nuclease-free water. The soaked animals were incubated at 20°C or 25°C for 24 hours in the thermocycler. 20°C incubation temperature was used for the DG4269 (*tn1753[gfp::3xflag::mex-3]*) strain while the 25°C temperature was used for the WRM24 (*sprSi17 [mex-5p*::*MODC PEST*::*GFP*::*H2B*::*mex-3 3´UTR + Cbr-unc-119(+)] II*) strain. After 24 hours, animals were placed on NGM plates seeded with *E*. *coli* OP50 and placed in the incubator. Once the animals reached adulthood, they were mounted on a 2% agarose pad on microscope slides, treated with 1mM levamisole to paralyze the animals, covered with a cover glass, then imaged.

### CRISPR/Cas9 mutagenesis

Ribonucleoprotein (RNP) mixes consisted of recombinant purified *Sp*Cas9 (final conc. = 2 μM), chemically synthesized crRNAs (final conc. = 40ng/μl) and tracrRNA (final conc. = 40ng/μl), commercial duplex buffer (30 mM HEPES, pH 7.5; 100 mM potassium acetate), and nuclease-free water. Sequences for the guide RNAs used are in S3 Table. *Sp*Cas9 was expressed from pET28a-Cas9-His (Addgene plasmid number 98158) and purified in our lab. The RNP mix was incubated at 37°C for 10 min. After the incubation, the plasmid pRF4 (*rol-6*) was added as a co-injection marker (final conc. = 50ng/μl). The mix was centrifuged at maximum speed for 5 min prior to loading a pulled borosilicate glass capillary injection needle. Young adult animals were microinjected in their gonads with the injection mix and then allowed to recover in M9 buffer on *E*. *coli* OP50 seeded NGM plates. The progeny of the injected animals was screened for the presence of roller animals, indicating a successful injection. All roller animals were singled out onto individual NGM plates, allowed to lay eggs, then lysed in a lysis buffer (30 mM Tris pH = 8, 8 mM EDTA, 100 mM NaCl, 0.7% NP-40, 0.7% Tween-20 + proteinase K just prior to use). Lysates were frozen at -80°C for at least 10 min, then incubated at 65°C for 1 hour and 95°C for 15 min prior to genotyping PCR. For a 25μl PCR reaction, 2μl of the lysate was used as a template. The primers used to detect *mex-3* 3´UTR deletions were (forward primer: 5´-GGCGGAAACATGAATCTGAGCCC- 3´, reverse primer: 5´-CGGACAATTGATCGGCCAATTGAC-3´). PCR reactions were run on a 1.5% TAE agarose gel. Single bands that are shorter than the wild type band indicate a homozygous mutation while two bands including the wild type band indicate a heterozygous mutation. Sanger sequencing of the purified PCR product was used to define the identity of the specific deletion.

### Poly(A) tail assay and TOPO cloning

N2, DG4269 (GFP::MEX-3), and all *mex-3* mutant animals were collected and washed in M9 buffer 6 times then frozen in trizol and stored at -80°C. Total RNA was isolated from these animals using phenol-chloroform and isopropanol extraction. For the poly(A) tail assay, a poly(A) tail assay kit (ThermoFisher Scientific cat #: 764551KT) was used following the protocol outlined by the manufacturer. For the tail-specific primer set, a universal reverse primer provided in the kit was used for all the strains. For N2, DG4269, *mex-3(spr5)*, *mex-3(spr6)*, and *mex-3(spr10)*, the forward primer 5´-CTACGCACAACTAACGGAGA-3´ was used. For *mex-3(spr9)*, the forward primer 5´-TCATGTCCTCCCTCAAAGG-3´ was used and for *mex-3(spr7)*, the forward primer 5´-CCCCAATATATATTCCTACAGTAGG-3´ was used. The PCR products were purified using a Zymo Research DNA clean and concentrator kit (cat #: D4034). The PCR products were cloned into a pCR4-TOPO TA vector using a TOPO TA Cloning kit (ThermoFisher Scientific cat #: K4575J10) following the manufacturer´s protocol. Plasmids containing the insert were analyzed using Sanger sequencing.

### Fluorescence microscopy

All of the imaging was done using a Zeiss Axioskop 2 plus microscope. ImageJ version 1.49 was used to quantify the images of the fluorescent animals from the RNAi experiments in the DG4269, WRM24, and the *mex-3* 3´UTR deletion mutant strains. For each animal, a line (width = 30 pixels) was drawn starting from the distal tip of the germline spanning the entire germline to the last oocyte. The fluorescence intensity was measured for each pixel in the line and then binned (total number = 20 bins for the DG4269 animals, 10 bins for the WRM24 animals). The fluorescence intensity from each animal was averaged across each bin. GraphPad Prism 7.04 was used to graph the mean fluorescence intensity for all the animals.

### Brood size

For each biological replicate, ~25 individual L3/L4 animals were placed on individual NGM plates seeded with *E*. *coli* OP50. Each animal was moved to a fresh plate after two days initially, and then moved again daily until the completion of the experiment. The number of eggs and larvae on the plate, from which the animal was moved, was counted 1–2 days later. The number of progeny is the total number of eggs and larvae produced during the animal´s fertile period. All animals were grown and counted at 20°C. N2 wild type animals were used as the control. Each assay consisted of three biological replicates.

### RNA-seq sample and library preparation

N2, *mex-3(spr9)*, and *mex-3(spr10)* animals were synchronized and then grown at 20°C. Six biological replicates for N2, four biological replicates for *mex-3(spr9)*, and three biological replicates for *mex-3(spr10)* were used for RNA-seq library preparations. For each biological replicate, 1–2 plates of young adults were washed with M9 buffer six times before addition of trizol. The animals in trizol were placed in the -80°C freezer prior to RNA isolation using phenol-chloroform and isopropanol precipitation. Ribosomal RNA was depleted using a rRNA depletion protocol [78]. Concentration of the RNA was measured using Qubit and then used for RNA-seq library preparation. RNA-seq library prep was performed using NEBNext Ultra II RNA library prep (cat# E7775S) following the manufacturer’s protocol. NEBNext Multiplex Oligos for IlIumina (Dual Index Primer Set 1) (cat# E7600) was used for library indexing. Concentration of the libraries was determined using both Qubit and fragment bioanalyzer. Libraries were pooled at a final concentration of 4nM prior to sequencing. Barcoded libraries were sequenced on an Ilumina NEXTSeq 500.

### Data analysis

Brood size data were analyzed using both Mann-Whitney U test and Kolmogorov-Smirnov nonparametric tests to compare the distributions between mutant and control strains. The data presented in each brood size assay represent a global analysis from three independent biological replicates. The p-values reported in Figs 2 and 3 are from the Kolmogorov-Smirnov test. In the imaging studies, a two tailed student t-test was used to compare the mean fluorescence intensities. For RNAi conditions that were compared to the same control data, an unstacked one-way ANOVA was used to assess the overall significance. Post-hoc pairwise p-values were calculated using the Fisher´s LSD test then corrected for multiple hypotheses using a Bonferroni adjustment by multiplying the p-values by the number of hypotheses tested. To analyze the nuclear fluorescence intensity in the *oma-1/2* RNAi animals and controls, a circle with a radius of 15 pixels was drawn in the nucleus and another circle of the same radius drawn in the cytoplasm of the same oocyte. We calculated the ratio by dividing the nuclear fluorescence intensity by the cytoplasmic fluorescence intensity. We calculated the ratios for the two most proximal oocytes and then averaged the two ratios for each individual animal. A two-tailed student t-test was used to compare the ratios of the control and treated animals. The RNA-seq analysis was performed using OneStopRNAseq online tool v1.0.0 [79]. MultiQC [80] was used to assess raw read quality while QoRTs was used to assess quality for post-alignment [81]. Reads were aligned to the reference genome assembly WBcel235 using star_2.7.5a [82] and annotated with WBcel235.90 [83]. Aligned exons were counted with featureCounts_2.0.0 [84]. Reads that mapped to the *mex-3* 3´UTR were not counted in the feature count analysis. Differential expression analysis was performed with DESeq2_1.28.1 [85]. ashR was used to create log2 fold change (LFC) shrinkage for each comparison. Genes were considered significantly differentially expressed if they had an FDR < 0.05 and absolute log2 fold change > 0.585. Wormbase was used for the tissue enrichment analysis [86]. WormCat was used to determine gene categories of the significantly differentially expressed genes [87].

### Prediction of putative MEX-3 targets

The predicted MEX-3 binding affinity was calculated for each possible site in annotated *C*. *elegans* 3’UTRs using a position weight matrix derived from previously published *in vitro* mutagenesis data [16]. The calculations make the assumption that recognition of each nucleotide recognized by MEX-3 is independent of its neighbors. The relative estimated K_d_ (compared to the best measured K_d_) was calculated using the formula K_rel_ = RT ln(ΔΔG°). 1/K_rel_ was plotted as a function of UTR position to generate the wiggle plots (S5 Fig), wherein a value of 1 indicates predicted binding equivalent to the control sequence, and values approaching zero indicating little to no propensity for MEX-3 interaction.

## Supporting information

S1 Fig Conservation of the *mex-3* 3´UTR in *C*. *elegans* and other nematodes. The *mex-3* 3´UTR contains homologues in *C*. *brenneri*, *C*. *remanei*, *C*. *briggsae*, and *C*. *janponic*a. Highlighted in light blue are the most conserved regions based on analysis by PhyloP on the UCSC genome browser.
(TIF)

S2 Fig View of the three most conserved regions in the *mex-3* 3´UTR and other *Caenorhabditis* nematodes. None of the conserved motifs in any of the three regions correspond to a candidate RNA-binding protein binding motif.
(TIF)

S3 Fig Summary of the expression pattern, binding specificity, and predicted binding motifs for GLD-1, LIN-41, OMA-1/2. (TIF)

S4 Fig DAZ-1, GLD-1, OMA-1/2, and LIN-41 regulate spatiotemporal expression pattern of MEX-3. **(A)** DIC and fluorescence images of wild type GFP::MEX-3 animals from the control RNAi. **(B)** DIC and fluorescence images of GFP::MEX-3 animals after *daz-1* knockdown. GFP::MEX-3 was significantly increased in the mitotic distal end. **(C)** DIC and fluorescence images of GFP::MEX-3 animals after *gld-1* knockdown. GFP::MEX-3 expression was derepressed in the meiotic region. **(D)** DIC and fluorescence images of GFP::MEX-3 animals after *lin-41* knockdown. GFP::MEX-3 was de-repressed in the loop region. **(E)** DIC and fluorescence images of GFP::MEX-3 animals after *oma-1/2* knockdown. GFP::MEX-3 was significantly increased in the oocytes. **(F)** quantitative analysis of fluorescence intensity after *daz-1* knockdown (n = 9/15). For all the images from the RNAi, a line with a width of 30 pixels was drawn along the entire germline and fluorescence intensities were binned (20 bins). Data are shown as the mean fluorescence intensity ± standard deviation (SD). A two tailed student t-test was performed to compare the means for each bin from control animals and the RNAi condition to assess significance. For RNAi conditions that have the same control, a one-way unstacked ANOVA was used to assess the overall significance, then Bonferroni adjusted p-values were calculated by multiplying pairwise Fisher´s LSD test p-values by the number of hypotheses tested. All p-values for this figure are reported in S4 Table. **(G)** quantitative analysis of fluorescence intensity after *gld-1* knockdown (n = 7/13). **(H)** quantitative analysis of fluorescence intensity after *lin-41* knockdown (n = 17/17). **(I)** quantitative analysis of fluorescence intensity after *oma-1/2* knockdown (n = 9/9). **(J)** quantitative analysis of nuclear GFP::MEX-3 of the *oma-1/2* RNAi animals. Nuclear fluorescence intensity was divided by the cytoplasmic fluorescence intensity for each oocyte. Each dot represents the averaged ratios from the two most proximal oocytes in an individual animal. (*) indicates statistical significance, adjusted p-value ≤ 0.05. (****) indicates statistical significance, p-value ≤ 0.0005. Scale bar = 30 μm.
(TIF)

S5 Fig Genome browser view of MEX-3 binding propensity to the 3´UTR of two of its known targets (*pal-1*, *nos-2*) and the seven differentially expressed genes in both *mex-3(spr9)* and *mex-3(spr10)* mutant animals. The wiggle plots show the predicted MEX-3 binding affinity in the 3´UTR. 1 indicates binding equivalent to the control sequence used to generate these plots while 0 indicates little or no binding affinity. The blue box highlights a motif that has a perfect match to the predicated MEX-3 recognition element (MRE).
(TIF)

S1 Table List of strains used in this paper. (DOCX)

S2 Table *In vitro* transcription primers for RNAi. (DOCX)

S3 Table Guide RNAs used to target the *mex-3* 3´UTR using CRISPR/Cas9. (DOCX)

S4 Table Student t-test p-values for bin to bin pairwise comparisons in the *mex-3* 3´UTR deletion mutants. (DOCX)

S5 Table Student t-test p-values for bin to bin pairwise comparisons of mean fluorescence intensity in the *mex-3* 3´UTR transgenic reporter strain. (DOCX)

S6 Table P-values for bin to bin pairwise comparisons of mean fluorescence intensity in the GFP::MEX-3 strain. Adjusted p-values for *gld-1*, *daz-1*, and *lin-41* are corrected for multiple hypothesis testing as described in the methods, while p-values for *oma-1/2* are from a student t-test.
(DOCX)

S7 Table P-values for bin to bin pairwise comparisons of mean fluorescence intensity in the GFP::MEX-3 strain. Adjusted p-values for *ccf-1*, *ntl-1*, and *ife-3* are corrected for multiple hypothesis testing as described in the methods, while p-values for *gld-2* and *gld-3* are from a student t-test.
(DOCX)

S8 Table Student t-test p-values for bin to bin pairwise comparisons of mean fluorescence intensity in the *mex-3* 3´UTR transgenic reporter strain. Adjusted p-values for *gld-2*, *gld-3*, and *ccf-1* are corrected for multiple hypothesis testing as described in the methods, while p-values for *ntl-1* are from a student t-test.
(DOCX)

S9 Table The length of the *mex-3* 3´UTR from sequenced TOPO cloning plasmids containing tail-specific PCR products from the poly(A) tail assay. Each number represents a single plasmid sequenced.
(DOCX)

S10 Table List of the differentially expressed genes between *mex-3(spr9*) and N2 or *mex-3(spr10)* and N2. (XLSX)

S11 Table List of the P-values from the tissue enrichment analysis and gene category analysis. (XLSX)
