# Supplementary figures and images for: The endogenous mex-3 3´UTR is required for germline repression and contributes to optimal fecundity in C. elegans
Source: PLoS Genet. 2021 Aug 23;17(8):e1009775. doi: 10.1371/journal.pgen.1009775 (PMC8412283; doi:10.1371/journal.pgen.1009775)

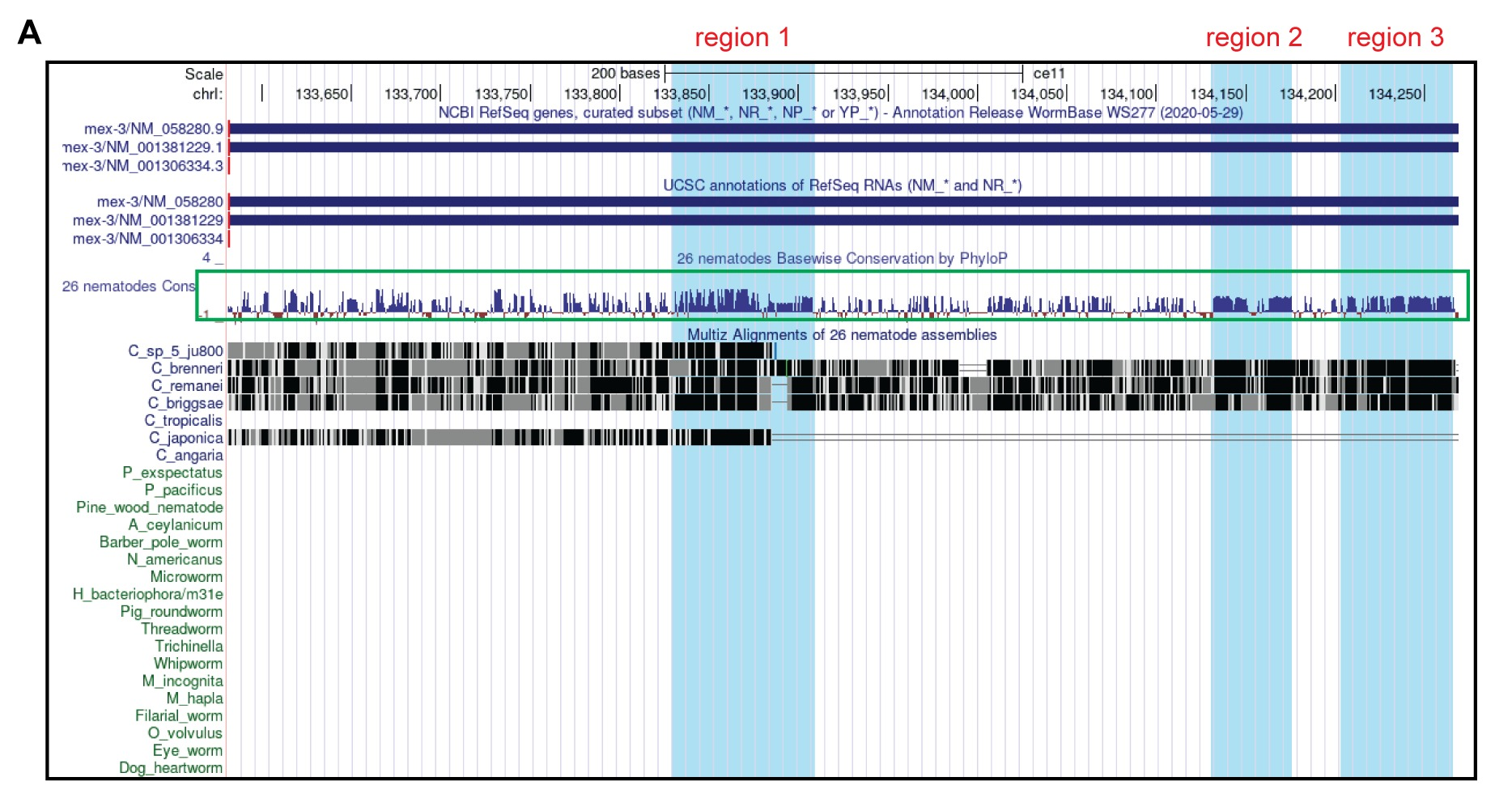

Supplement: S1 Fig — The mex-3 3´UTR contains homologues in C. brenneri, C. remanei, C. briggsae, and C. janponica. Highlighted in light blue are the most conserved regions based on analysis by PhyloP on the UCSC genome browser. (TIF) [file pgen.1009775.s001.tif]

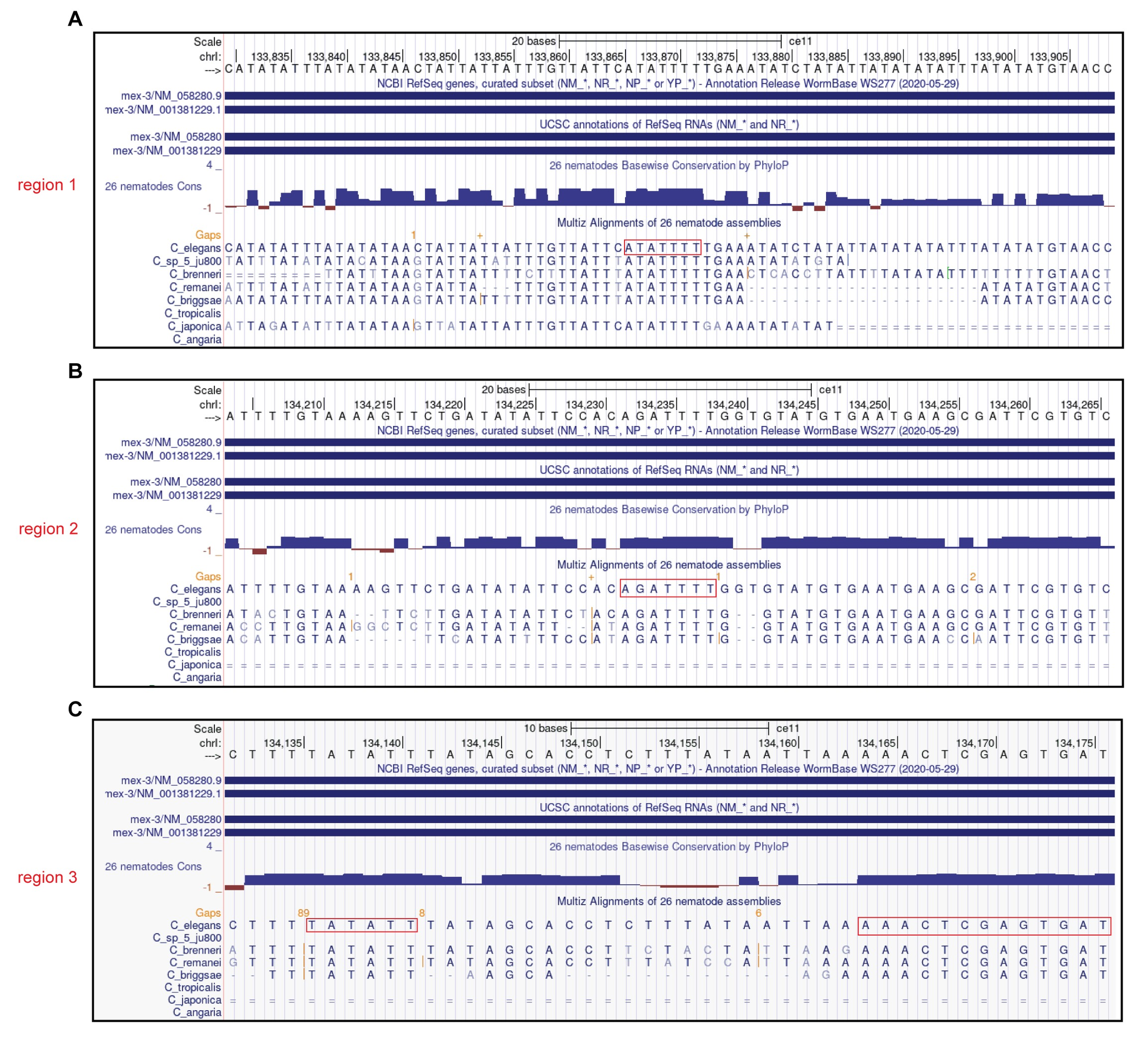

Supplement: S2 Fig — None of the conserved motifs in any of the three regions correspond to a candidate RNA-binding protein binding motif. (TIF) [file pgen.1009775.s002.tif]

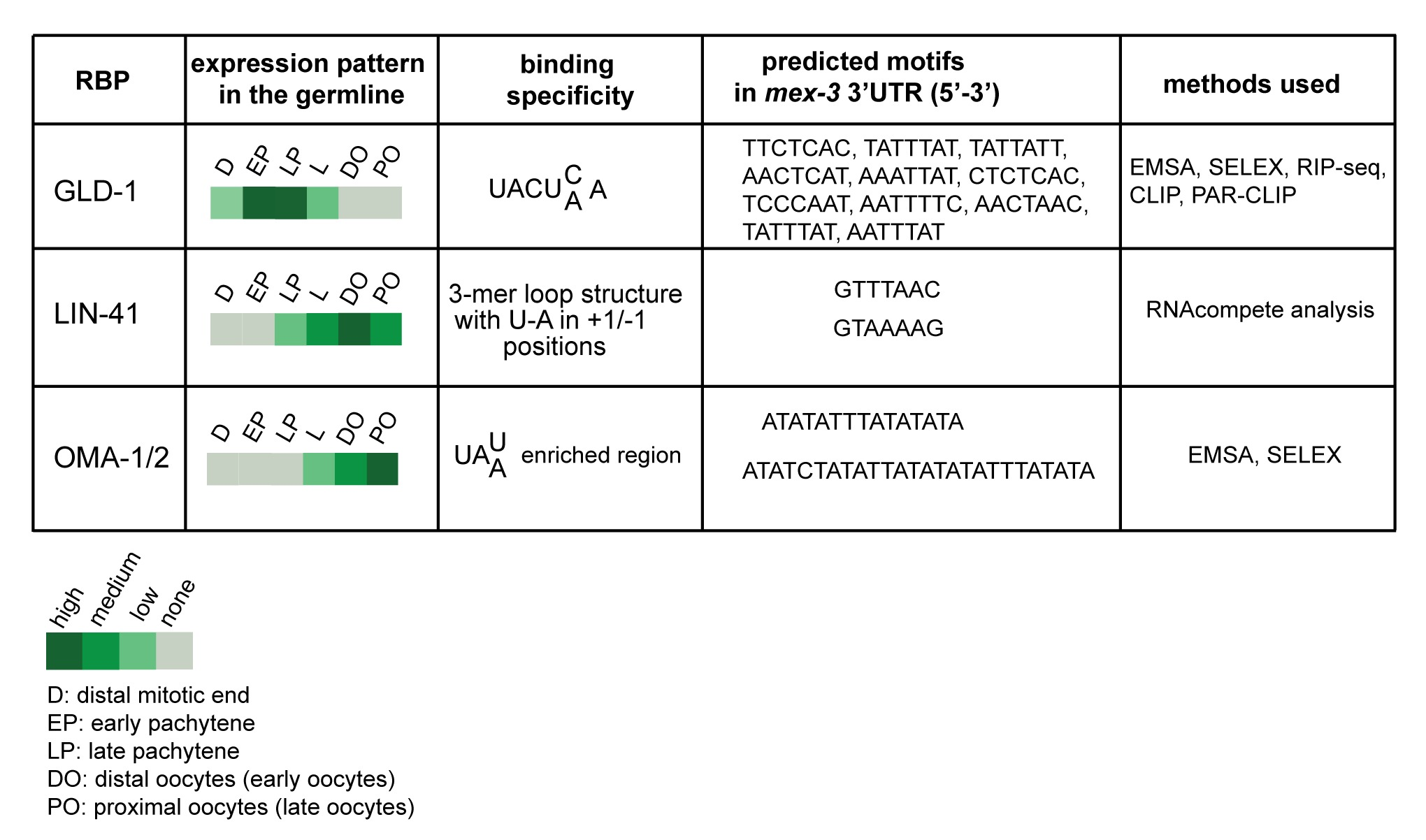

Supplement: S3 Fig — (TIF) [file pgen.1009775.s003.tif]

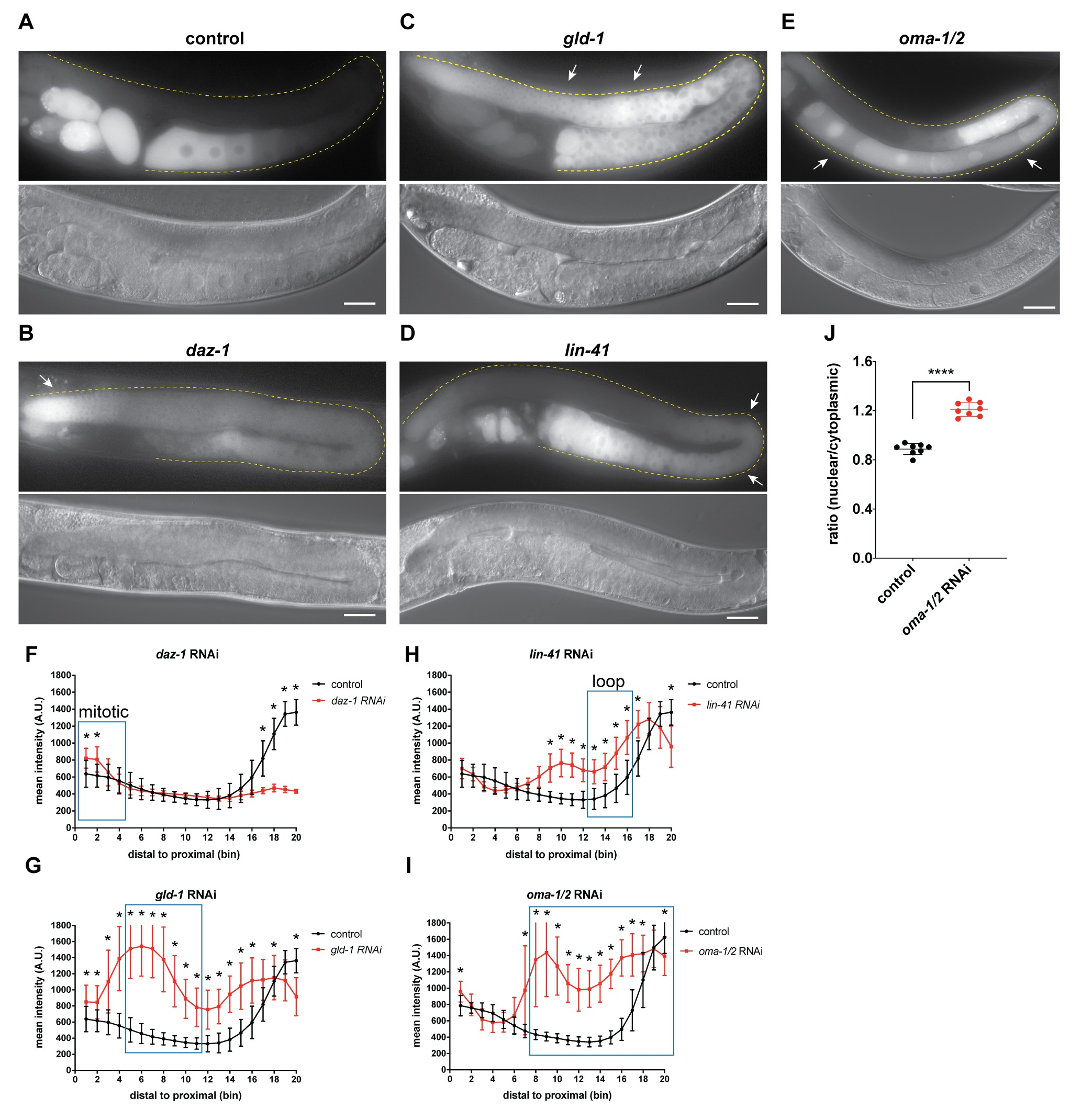

Supplement: S4 Fig — (A) DIC and fluorescence images of wild type GFP::MEX-3 animals from the control RNAi. (B) DIC and fluorescence images of GFP::MEX-3 animals after daz-1 knockdown. GFP::MEX-3 was significantly increased in the mitotic distal end. (C) DIC and fluorescence images of GFP::MEX-3 animals after gld-1 knockdown. GFP::MEX-3 expression was derepressed in the meiotic region. (D) DIC and fluorescence images of GFP::MEX-3 animals after lin-41 knockdown. GFP::MEX-3 was de-repressed in the loop region. (E) DIC and fluorescence images of GFP::MEX-3 animals after oma-1/2 knockdown. GFP::MEX-3 was significantly increased in the oocytes. (F) quantitative analysis of fluorescence intensity after daz-1 knockdown (n = 9/15). For all the images from the RNAi, a line with a width of 30 pixels was drawn along the entire germline and fluorescence intensities were binned (20 bins). Data are shown as the mean fluorescence intensity ± standard deviation (SD). A two tailed student t-test was performed to compare the means for each bin from control animals and the RNAi condition to assess significance. For RNAi conditions that have the same control, a one-way unstacked ANOVA was used to assess the overall significance, then Bonferroni adjusted p-values were calculated by multiplying pairwise Fisher´s LSD test p-values by the number of hypotheses tested. All p-values for this figure are reported in S4 Table. (G) quantitative analysis of fluorescence intensity after gld-1 knockdown (n = 7/13). (H) quantitative analysis of fluorescence intensity after lin-41 knockdown (n = 17/17). (I) quantitative analysis of fluorescence intensity after oma-1/2 knockdown (n = 9/9). (J) quantitative analysis of nuclear GFP::MEX-3 of the oma-1/2 RNAi animals. Nuclear fluorescence intensity was divided by the cytoplasmic fluorescence intensity for each oocyte. Each dot represents the averaged ratios from the two most proximal oocytes in an individual animal. (*) indicates statistical significance, adjusted p-value ≤ [file pgen.1009775.s004.tif]

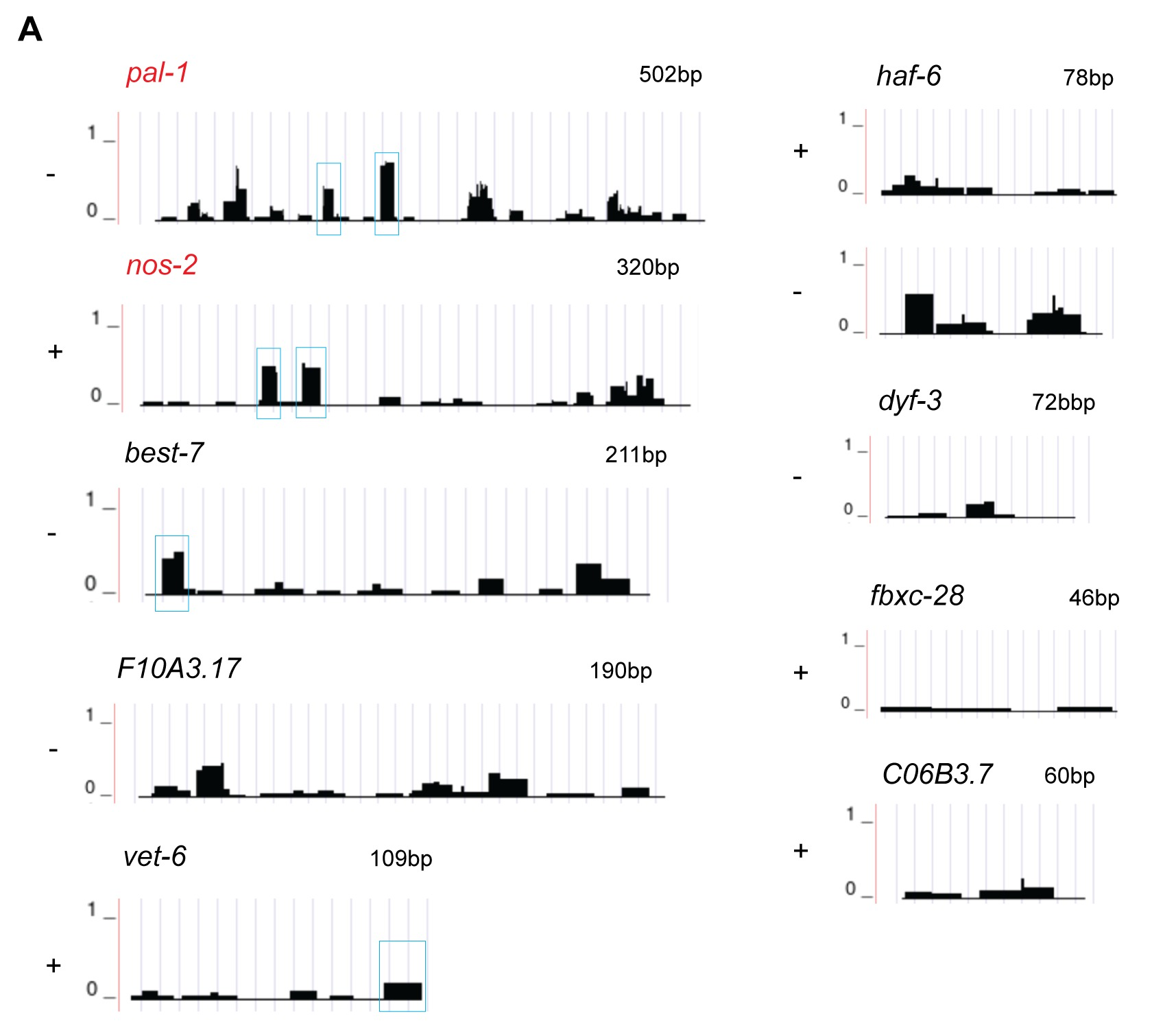

Supplement: S5 Fig — The wiggle plots show the predicted MEX-3 binding affinity in the 3´UTR. 1 indicates binding equivalent to the control sequence used to generate these plots while 0 indicates little or no binding affinity. The blue box highlights a motif that has a perfect match to the predicated MEX-3 recognition element (MRE). (TIF) [file pgen.1009775.s005.tif]
