# Supplementary material for: The endogenous mex-3 3´UTR is required for germline repression and contributes to optimal fecundity in C. elegans
Source: PLoS Genet. 2021 Aug 23;17(8):e1009775. doi: 10.1371/journal.pgen.1009775 (PMC8412283; doi:10.1371/journal.pgen.1009775)
Supplement: S1 Table — (DOCX) [file pgen.1009775.s006.docx]

**S1 Table. List of strains used in this paper**

| **Strain** | **Description** |
| --- | --- |
| N2 | wild type |
| DG4269 | *mex-3(tn1753[gfp::3xflag::mex-3]) I* |
| WRM45 | *mex-3(spr5) I* |
| WRM49 | *mex-3(spr6) I* |
| WRM50 | *mex-3(spr7) I* |
| WRM52 | *mex-3(spr9) I* |
| WRM53 | *mex-3(spr10) I* |
| WRM24 | sprSi17 [mex-5p::MODC PEST::GFP::H2B::mex-3 3´UTR + Cbr-unc-119(+)] II |
