# Supplementary material for: The endogenous mex-3 3´UTR is required for germline repression and contributes to optimal fecundity in C. elegans
Source: PLoS Genet. 2021 Aug 23;17(8):e1009775. doi: 10.1371/journal.pgen.1009775 (PMC8412283; doi:10.1371/journal.pgen.1009775)
Supplement: S2 Table — (DOCX) [file pgen.1009775.s007.docx]

**S2 Table.** ***In vitro* transcription primers for RNAi**

| **Name** | **Sequence** |
| --- | --- |
| gld-1frag1F | 5´-ATGCCGTCGTGCACCACT-3´ |
| gld-1frag1R | 5´-CAATTGGCCTTTCCACGGTG-3´ |
| gld-2frag1F | 5´-GCAGAACGAAACGACGAACAC-3´ |
| gld-2frag1R | 5´-ATTGGATCCTGCCCGCGT-3´ |
| gld-3frag1F | 5´-ATGGGGGAGCAAAGCCATG-3´ |
| gld-3frag1R | 5´-TCCGGTGCCGGAGACTCG-3´ |
| oma-1frag1F | 5´-ATGAACGTTAACGGTGAAAACAACG-3´ |
| oma-1frag1R | 5´-GCGAGACGGTGGATAGGTCATC-3´ |
| oma-2frag1F | 5´-ATGGATATGCTCAAGGAAAATGTTATCC-3´ |
| oma-2frag1R | 5´-GTTGATGGATCCAACGGCCAG-3´ |
| lin-41frag1F | 5´- ATCGTGCCATGCTCATTGGAG-3´ |
| lin-41frag1R | 5´- GGAATTACGTGGTGGAGCTATGG-3´ |
| lin-41frag2F | 5´- GATGGCTACTTTGATGAGCCGT-3´ |
| lin-41frag2R | 5´- CTCACGGGGTGTCATTGTGAC-3´ |
| daz-1frag1F | 5´- ATGTCGCCGCCTCTACGGTATC-3´ |
| daz-1frag1R | 5´- CTGCTGCTGCTGCACATTGG-3´ |
| ccr-4fragF | 5´-GACAGTGGAGACGGCGAATC-3´ |
| ccr-4fragR | 5´-CCGGTGGAGGATGAGTGTTAACATG-3´ |
| ccf-1frag1F | 5´-ATGGCTTCTAGTAGCAGTGGTGG-3´ |
| ccf-1frag1R | 5´-GTCCACAGATCAATGGAGCAATCT-3´ |
| ntl-1fragF | 5´-CTCCAAAAAAGGCACCATCACGC-3´ |
| ntl-1fragR | 5´-CGGCGAATTATCCTGTCTTCCATTTTC-3´ |
| ntl-1frag2F | 5´-GGTGTGGAATGATACCAGCCTTTCAAAAC-3´ |
| ntl-1frag2R | 5´-GCCAGAGTTTGAATTCTGCCGTTGAGTAAG-3´ |
| ife-3fragF | 5´- GGGAGGATTGTCTGAAGATGGTTTCACTTTTCG-3´ |
| ife-3fragR | 5´- GAGATCTTTAGAATATGCTTAAGGAGTTGGGG-3´ |
