# Supplementary material for: The endogenous mex-3 3´UTR is required for germline repression and contributes to optimal fecundity in C. elegans
Source: PLoS Genet. 2021 Aug 23;17(8):e1009775. doi: 10.1371/journal.pgen.1009775 (PMC8412283; doi:10.1371/journal.pgen.1009775)
Supplement: S3 Table — (DOCX) [file pgen.1009775.s008.docx]

**S3 Table.** **Guide RNAs used to target the *mex-3* 3´UTR using CRISPR/Cas9**

| **Name** | **Sequence** |
| --- | --- |
| mex-3cr1 | 5´-GAGAGTCTACACGATAGTAA-3´ |
| mex-3cr2 | 5´-TATATATTGGGGGACTGCAT-3´ |
| mex-3cr3 | 5´-TAGTTGTGCGTAGTAGAGAG-3´ |
| mex-3cr4 | 5´-TTCACATACACCAAAATCTG-3´ |
| Mex-3cr5 | 5’-CCATTTTCTACTTTGTTCAT-3’ |
