# Supplementary material for: The endogenous mex-3 3´UTR is required for germline repression and contributes to optimal fecundity in C. elegans
Source: PLoS Genet. 2021 Aug 23;17(8):e1009775. doi: 10.1371/journal.pgen.1009775 (PMC8412283; doi:10.1371/journal.pgen.1009775)
Supplement: S4 Table — (DOCX) [file pgen.1009775.s009.docx]

**S4 Table. Student t-test p-values for bin to bin pairwise comparisons in the *mex-3* 3´UTR deletion mutants in figures 2 and 3**

| **bin #** | ***mex-3(spr6)*** | **Fold change** | ***mex-3(spr7)*** | **Fold change** | ***mex-3(spr9)*** | **Fold change** | ***mex-3(spr10)*** | **Fold change** |
| --- | --- | --- | --- | --- | --- | --- | --- | --- |
| 1 | 0.8821 | 1.01 | 0.6112 | 0.97 | 0.0163 | 1.18 | 0.238 | 0.93 |
| 2 | 0.6463 | 0.97 | 0.5451 | 0.95 | 0.0304 | 1.15 | 0.0374 | 0.87 |
| 3 | 0.9121 | 0.99 | 0.5995 | 0.96 | 0.0028 | 1.24 | 0.0633 | 0.88 |
| 4 | 0.7874 | 1.02 | 0.9839 | 1.00 | 4.91E-07 | 1.47 | 0.5389 | 0.96 |
| 5 | 0.6907 | 1.03 | 0.2443 | 1.09 | 3.87E-11 | 1.80 | 0.3872 | 1.05 |
| 6 | 0.3152 | 1.06 | 0.0452 | 1.14 | 0 | 2.17 | 0.159 | 1.07 |
| 7 | 0.1001 | 1.10 | 0.016 | 1.14 | 0 | 2.56 | 0.1092 | 1.08 |
| 8 | 0.0393 | 1.11 | 0.0159 | 1.14 | 0 | 2.87 | 0.065 | 1.10 |
| 9 | 0.0239 | 1.13 | 0.043 | 1.13 | 0 | 2.98 | 0.0671 | 1.11 |
| 10 | 0.0164 | 1.13 | 0.1226 | 1.12 | 0 | 3.01 | 0.0503 | 1.12 |
| 11 | 0.0102 | 1.13 | 0.2614 | 1.09 | 0 | 2.98 | 0.0494 | 1.13 |
| 12 | 0.0094 | 1.15 | 0.5356 | 1.04 | 0 | 2.80 | 0.0345 | 1.17 |
| 13 | 0.0157 | 1.15 | 0.8783 | 1.01 | 9.11E-11 | 2.48 | 0.017 | 1.24 |
| 14 | 0.0899 | 1.12 | 0.3192 | 0.95 | 2.28E-10 | 2.27 | 0.0218 | 1.28 |
| 15 | 0.0684 | 1.14 | 0.0328 | 0.88 | 1.26E-11 | 2.21 | 0.0265 | 1.35 |
| 16 | 0.0912 | 1.12 | 0.0015 | 0.81 | 7.52E-12 | 2.04 | 0.0383 | 1.32 |
| 17 | 0.0669 | 1.12 | 0.0004 | 0.78 | 7.52E-11 | 1.72 | 0.0817 | 1.20 |
| 18 | 0.0204 | 1.14 | 0.0016 | 0.83 | 2.11E-06 | 1.37 | 0.23 | 1.10 |
| 19 | 0.0382 | 1.12 | 0.0091 | 0.87 | 0.1166 | 1.10 | 0.8602 | 1.01 |
| 20 | 0.0498 | 1.11 | 0.0846 | 0.90 | 0.8395 | 0.99 | 0.6583 | 0.97 |
