# Supplementary material for: The endogenous mex-3 3´UTR is required for germline repression and contributes to optimal fecundity in C. elegans
Source: PLoS Genet. 2021 Aug 23;17(8):e1009775. doi: 10.1371/journal.pgen.1009775 (PMC8412283; doi:10.1371/journal.pgen.1009775)
Supplement: S5 Table — (DOCX) [file pgen.1009775.s010.docx]

**S5 Table. Student t-test p-values for bin to bin pairwise comparisons of mean fluorescence intensity in the *mex-3* 3´UTR transgenic reporter strain in figure 4**

| **bin #** | ***gld-1*** | **Fold change** | ***daz-1*** | **Fold change** | ***lin-41*** | **Fold change** | ***oma-1/2*** | **Fold change** |
| --- | --- | --- | --- | --- | --- | --- | --- | --- |
| 1 | 0.1206 | 1.14 | 0.1026 | 0.91 | 0.0127 | 0.78 | 4.68E-05 | 0.80 |
| 2 | 0.0674 | 1.27 | 0.3354 | 0.94 | 0.2065 | 0.88 | 1.06E-01 | 0.93 |
| 3 | 0.0348 | 1.56 | 0.9630 | 1.00 | 0.8094 | 0.98 | 1.77E-02 | 1.17 |
| 4 | 0.0043 | 1.83 | 0.0415 | 1.08 | 0.7648 | 0.97 | 9.86E-06 | 1.68 |
| 5 | 0.0061 | 1.75 | 0.0803 | 1.08 | 0.4667 | 1.07 | 1.38E-12 | 2.10 |
| 6 | 0.0080 | 1.66 | 0.4952 | 1.04 | 0.1113 | 1.15 | 1.35E-12 | 2.25 |
| 7 | 0.0122 | 1.55 | 0.6957 | 1.03 | 0.0054 | 1.39 | 5.22E-10 | 2.20 |
| 8 | 0.0070 | 1.55 | 0.6804 | 0.97 | 0.0150 | 1.53 | 3.05E-12 | 2.17 |
| 9 | 0.0922 | 1.19 | 0.0048 | 0.83 | 0.0168 | 1.59 | 6.19E-05 | 1.52 |
| 10 | 0.0060 | 0.66 | 1.34E-09 | 0.43 | 0.7647 | 1.05 | 4.49E-01 | 1.07 |
