# Supplementary material for: The endogenous mex-3 3´UTR is required for germline repression and contributes to optimal fecundity in C. elegans
Source: PLoS Genet. 2021 Aug 23;17(8):e1009775. doi: 10.1371/journal.pgen.1009775 (PMC8412283; doi:10.1371/journal.pgen.1009775)
Supplement: S6 Table — Adjusted p-values for gld-1, daz-1, and lin-41 are corrected for multiple hypothesis testing as described in the methods, while p-values for oma-1/2 are from a student t-test. (DOCX) [file pgen.1009775.s011.docx]

**S6 Table. P-values for bin to bin pairwise comparisons of mean fluorescence intensity in the GFP::MEX-3 strain in figure S3.** Adjusted p-values for *gld-1*, *daz-1*, and *lin-41* are corrected for multiple hypothesis testing as described in the methods, while p-values for *oma-1/2* are from a student t-test.

| **bin #** | ***gld-1*** | **Fold change** | ***daz-1*** | **Fold change** | ***lin-41*** | **Fold change** | ***oma-1/2*** | **Fold change** |
| --- | --- | --- | --- | --- | --- | --- | --- | --- |
| 1 | 0.012 | 1.33 | 0.022 | 1.29 | 1.408 | 1.10 | 1.42E-02 | 1.22 |
| 2 | 0.004 | 1.37 | 0.012 | 1.31 | 4.304 | 1.03 | 0.4818 | 1.05 |
| 3 | 0.000 | 1.85 | 2.310 | 1.10 | 0.319 | 0.81 | 0.0598 | 0.85 |
| 4 | 0.000 | 2.50 | 3.838 | 0.95 | 0.151 | 0.78 | 0.0574 | 0.84 |
| 5 | 0.000 | 3.00 | 3.012 | 0.92 | 1.793 | 0.89 | 0.5329 | 0.94 |
| 6 | 0.000 | 3.37 | 4.385 | 0.96 | 3.805 | 1.05 | 0.1734 | 1.23 |
| 7 | 0.000 | 3.62 | 5.914 | 1.00 | 0.457 | 1.26 | 0.0217 | 2.05 |
| 8 | 0.000 | 3.52 | 5.046 | 1.05 | 0.054 | 1.54 | 4.50E-04 | 3.13 |
| 9 | 0.000 | 3.02 | 4.476 | 1.08 | 0.000 | 1.92 | 8.00E-05 | 3.53 |
| 10 | 0.000 | 2.57 | 4.081 | 1.12 | 0.000 | 2.21 | 4.86E-06 | 3.29 |
| 11 | 0.000 | 2.35 | 3.840 | 1.12 | 0.000 | 2.23 | 5.64E-07 | 2.92 |
| 12 | 0.001 | 2.29 | 4.657 | 1.08 | 0.000 | 2.05 | 7.15E-06 | 2.82 |
| 13 | 0.000 | 2.32 | 5.736 | 1.02 | 0.001 | 1.94 | 9.37E-07 | 2.91 |
| 14 | 0.000 | 2.47 | 4.611 | 0.92 | 0.001 | 1.88 | 4.37E-07 | 3.00 |
| 15 | 0.001 | 2.25 | 3.302 | 0.82 | 0.004 | 1.89 | 1.07E-08 | 2.96 |
| 16 | 0.000 | 1.88 | 0.607 | 0.68 | 0.000 | 1.79 | 8.06E-08 | 2.77 |
| 17 | 0.050 | 1.37 | 0.003 | 0.54 | 0.000 | 1.49 | 7.00E-05 | 1.93 |
| 18 | 4.490 | 1.04 | 0.000 | 0.42 | 0.628 | 1.16 | 0.0321 | 1.30 |
| 19 | 0.830 | 0.83 | 0.000 | 0.34 | 1.079 | 0.88 | 0.9039 | 0.99 |
| 20 | 0.016 | 0.67 | 0.000 | 0.32 | 0.003 | 0.70 | 0.0458 | 0.86 |
