# Supplementary material for: The endogenous mex-3 3´UTR is required for germline repression and contributes to optimal fecundity in C. elegans
Source: PLoS Genet. 2021 Aug 23;17(8):e1009775. doi: 10.1371/journal.pgen.1009775 (PMC8412283; doi:10.1371/journal.pgen.1009775)
Supplement: S7 Table — Adjusted p-values for ccf-1, ntl-1, and ife-3 are corrected for multiple hypothesis testing as described in the methods, while p-values for gld-2 and gld-3 are from a student t-test. (DOCX) [file pgen.1009775.s012.docx]

**S7 Table. P-values for bin to bin pairwise comparisons of mean fluorescence intensity in the GFP::MEX-3 strain in figures 5, 6, and 7.** Adjusted p-values for *ccf-1*, *ntl-1*, and *ife-3* are corrected for multiple hypothesis testing as described in the methods, while p-values for *gld-2* and *gld-3* are from a student t-test.

| **bin #** | ***gld-2*** | **Fold change** | ***gld-3*** | **Fold change** | ***ccf-1*** | **Fold change** | ***ntl-1*** | **Fold change** | ***ife-3*** | **Fold change** |
| --- | --- | --- | --- | --- | --- | --- | --- | --- | --- | --- |
| 1 | 0.0273 | 0.81 | 0.8979 | 0.99 | 0.000 | 1.44 | 0.017 | 1.27 | 2.216 | 0.92 |
| 2 | 0.0078 | 0.82 | 0.9459 | 1.00 | 0.000 | 1.41 | 0.910 | 1.13 | 3.698 | 0.96 |
| 3 | 0.0003 | 0.77 | 0.5631 | 1.05 | 3.116 | 1.06 | 1.031 | 0.86 | 4.769 | 0.97 |
| 4 | 0.0019 | 0.81 | 0.3981 | 1.10 | 1.920 | 0.90 | 0.112 | 0.76 | 1.585 | 1.11 |
| 5 | 0.1225 | 0.90 | 0.3508 | 1.10 | 4.571 | 0.97 | 0.312 | 0.79 | 0.069 | 1.28 |
| 6 | 0.8322 | 0.99 | 0.0953 | 1.14 | 1.148 | 1.15 | 0.573 | 0.81 | 0.004 | 1.41 |
| 7 | 0.4870 | 1.05 | 0.0164 | 1.19 | 0.007 | 1.50 | 1.530 | 0.82 | 0.007 | 1.52 |
| 8 | 0.4606 | 1.05 | 0.0217 | 1.22 | 0.004 | 1.73 | 2.695 | 0.84 | 0.039 | 1.61 |
| 9 | 0.7759 | 1.02 | 0.0308 | 1.25 | 0.001 | 1.87 | 3.123 | 0.85 | 0.028 | 1.68 |
| 10 | 0.9706 | 1.00 | 0.0172 | 1.24 | 0.001 | 2.06 | 3.460 | 0.85 | 0.012 | 1.85 |
| 11 | 0.8608 | 0.99 | 0.0329 | 1.27 | 0.000 | 2.00 | 2.320 | 0.79 | 0.001 | 1.92 |
| 12 | 0.8790 | 0.99 | 0.0082 | 1.51 | 0.004 | 1.89 | 2.344 | 0.78 | 0.005 | 1.90 |
| 13 | 0.8904 | 0.99 | 0.0053 | 2.00 | 0.000 | 2.12 | 2.638 | 0.80 | 0.047 | 1.69 |
| 14 | 0.6629 | 0.97 | 0.0032 | 2.55 | 0.000 | 1.98 | 4.166 | 0.91 | 0.070 | 1.61 |
| 15 | 0.1421 | 0.91 | 0.0020 | 2.84 | 0.003 | 1.93 | 4.947 | 0.94 | 0.224 | 1.57 |
| 16 | 0.0339 | 0.84 | 0.0009 | 2.78 | 0.198 | 1.36 | 1.284 | 0.78 | 0.367 | 1.33 |
| 17 | 0.0406 | 0.81 | 0.0002 | 2.36 | 4.466 | 1.04 | 0.073 | 0.71 | 4.487 | 1.04 |
| 18 | 0.4110 | 0.92 | 0.0003 | 1.77 | 0.539 | 0.83 | 0.052 | 0.72 | 0.222 | 0.78 |
| 19 | 0.9340 | 1.01 | 0.0010 | 1.42 | 0.001 | 0.64 | 0.138 | 0.78 | 0.008 | 0.69 |
| 20 | 0.9242 | 1.01 | 0.0367 | 1.18 | 0.000 | 0.63 | 0.494 | 0.84 | 0.001 | 0.66 |
