# Supplementary material for: The endogenous mex-3 3´UTR is required for germline repression and contributes to optimal fecundity in C. elegans
Source: PLoS Genet. 2021 Aug 23;17(8):e1009775. doi: 10.1371/journal.pgen.1009775 (PMC8412283; doi:10.1371/journal.pgen.1009775)
Supplement: S8 Table — Adjusted p-values for gld-2, gld-3, and ccf-1 are corrected for multiple hypothesis testing as described in the methods, while p-values for ntl-1 are from a student t-test. (DOCX) [file pgen.1009775.s013.docx]

**S8 Table. Student t-test p-values for bin to bin pairwise comparisons of mean fluorescence intensity in the *mex-3* 3´UTR transgenic reporter strain in figure 4.** Adjusted p-values for *gld-2*, *gld-3*, and *ccf-1* are corrected for multiple hypothesis testing as described in the methods, while p-values for *ntl-1* are from a student t-test

| **bin #** | ***gld-2*** | **fold change** | ***gld-3*** | **fold change** | ***ccf-1*** | **fold change** | ***ntl-1*** | **fold change** |
| --- | --- | --- | --- | --- | --- | --- | --- | --- |
| 1 | 0.017 | 0.83 | 0.217 | 0.88 | 1.884 | 1.07 | 0.00 | 0.72 |
| 2 | 0.006 | 0.84 | 0.513 | 0.93 | 0.959 | 1.09 | 0.00 | 0.69 |
| 3 | 0.014 | 0.86 | 1.731 | 0.97 | 0.077 | 1.12 | 0.00 | 0.79 |
| 4 | 0.329 | 0.93 | 1.757 | 1.03 | 0.008 | 1.14 | 0.00 | 0.89 |
| 5 | 2.083 | 0.98 | 0.139 | 1.10 | 0.024 | 1.12 | 0.01 | 0.91 |
| 6 | 2.214 | 0.98 | 0.051 | 1.14 | 0.042 | 1.13 | 0.34 | 0.94 |
| 7 | 1.323 | 0.95 | 0.062 | 1.16 | 0.291 | 1.09 | 0.46 | 0.95 |
| 8 | 1.288 | 0.93 | 0.001 | 1.41 | 2.671 | 1.00 | 0.97 | 1.00 |
| 9 | 0.021 | 0.72 | 0.000 | 1.70 | 0.145 | 0.79 | 0.09 | 0.81 |
| 10 | 0.000 | 0.31 | 0.327 | 1.08 | 0.000 | 0.37 | 0.00 | 0.40 |
