# Supplementary material for: The endogenous mex-3 3´UTR is required for germline repression and contributes to optimal fecundity in C. elegans
Source: PLoS Genet. 2021 Aug 23;17(8):e1009775. doi: 10.1371/journal.pgen.1009775 (PMC8412283; doi:10.1371/journal.pgen.1009775)
Supplement: S9 Table — Each number represents a single plasmid sequenced. (DOCX) [file pgen.1009775.s014.docx]

**S9 Table. The length of the *mex-3* 3´UTR from sequenced TOPO cloning plasmids containing tail-specific PCR products from the poly(A) tail assay. Each number represents a single plasmid sequenced**

| **strain** | **3´UTR Processing site** |
| --- | --- |
| N2 | 684/687 |
| DG4269 | 685 |
| *mex-3(spr5)* | 685/684 |
| *mex-3(spr6)* | 685/684 |
| *mex-3(spr7)* | 685/685 |
| *mex-3(spr9)* | 685/685/687 |
| *mex-3(spr10)* | 687/641 |
